# Behaviour-structure interplay drives acoustic signal divergence: emergence of multiple mechanisms in closely related crickets

**DOI:** 10.7717/peerj.21036

**Published:** 2026-06-10

**Authors:** Teddy Gaiddon, Augustin Lafond, Romain Nattier, Frédéric Legendre, Sandra Goutte, Karla Yotoko, Tony Robillard

**Affiliations:** 1 Institut de Systématique, Évolution, Biodiversité (ISYEB), Muséum National d’Histoire Naturelle, CNRS, Sorbonne Université, EPHE-PSL, Université des Antilles, Paris, France; 2 Marseille, France; 3 Division of Science, New York University Abu Dhabi, Abu Dhabi, United Arab Emirates; 4 Laboratório de Bioinformática e Evolução, Departamento de Biologia Geral, Universidade Federal de Viçosa, Viçosa, Brazil

**Keywords:** Acoustic communication, Signal evolution, Stridulation, Harmonic amplification, Crickets, Phylogenetic comparative methods

## Abstract

Acoustic communication plays a central role in reproductive isolation, yet the mechanisms driving signal divergence among closely related species remain poorly understood. In male crickets, calling songs emerge from the interaction between the morphology of the stridulatory apparatus and the behaviour controlling forewing movements. Species of the genus *Agnothecous* are morphologically similar in their sound-producing structures, yet emit high-frequency calls spanning approximately 10–20 kHz, making this group a suitable model to investigate this interaction. We analysed 15 species using acoustic recordings, morphological measurements of the stridulatory apparatus, behavioural estimates of forewing kinematics, and comparative phylogenetic methods. Two distinct mechanisms of high-frequency song production were identified. Most species rely on *harmonic amplification*, in which stridulation generates a low fundamental frequency while resonant forewings selectively amplify one of its harmonics. In contrast, *A. robustus* and *A. tapinopus* independently evolved a *high-speed stridulation* mechanism, producing dominant frequencies directly through accelerated forewing closure. Although both mechanisms generate similar acoustic outputs, they differ in their biomechanical basis. Phylogenetic reconstructions indicate that *harmonic amplification* is ancestral in *Agnothecous*, with *high-speed stridulation* evolving convergently in larger-bodied species. Bayesian correlation analyses across 11 continuous traits show that body size, stridulatory file, harp dimensions, and wing-closing speed form a tightly correlated trait complex that jointly shapes dominant frequency and syllable structure. Together, these results suggest energetic trade-offs and possible irreversibility of high-frequency communication. More broadly, they illustrate how morphological variation and behavioural plasticity interact to drive acoustic diversification in closely related species.

## Introduction

Acoustic communication signals are complex phenotypes composed of multiple semi-independent traits shaped by diverse selective pressures (Oh & Shaw, 2013). These pressures include intraspecific processes such as sexual selection, interactions between signal transmission and environmental conditions (sensory drive), and interspecific forces such as predation, parasitism, competition, or reinforcement (*e.g.*, Endler, 1992; Smith & Harper, 2003; Gonzalez-Voyer et al., 2013). Because acoustic signals play a central role in mate attraction and recognition, their divergence can promote reproductive isolation and ultimately drive speciation, both in the presence and absence of geographic barriers (allopatric or sympatric/parapatric speciation; Boughman, 2002; Slabbekoorn & Smith, 2002). Although divergence of calling signals between closely related species is widespread (Henry, 1994; Wilkins, Seddon & Safran, 2013), the mechanisms underlying this diversification remain insufficiently understood. Acoustic signals can generally be decomposed into elementary call units, defined as the smallest sound elements produced by the sound-generation system. Interspecific variation in calls arises from two main sources. First, calls may differ in their temporal organisation, such as rhythm or repetition rate, which is primarily controlled by behavioural traits mediated by specific neuronal circuits (*e.g.*, Gerhardt & Huber, 2002; Schöneich & Hedwig, 2012). Second, calls may differ in the properties of the call unit itself, which depend jointly on behavioural traits (how sound-producing structures are used) and morphological traits (the size, shape, and physical properties of these structures) (*e.g.*, in crickets: Bailey, 1970; Bennet-Clark, 1999). As a result, the evolution of call-unit characteristics cannot be understood by considering morphology or behaviour in isolation, but rather requires an integrated approach.

Despite increasing recognition of the tight coupling between morphology and behaviour in phenotypic evolution (Bertossa, 2011), disentangling their respective contributions remains challenging in acoustic communication systems. This is particularly true when sound-producing structures are internal or when functional mechanisms require detailed physiological or biomechanical characterisation. These difficulties are further amplified in a comparative and phylogenetic context, where similar signal phenotypes may arise through distinct evolutionary pathways.

Crickets provide a well-established model to investigate these issues (*e.g.*, Huber, 1962; Hoy & Paul, 1973; Schildberger, 1994; Gerhardt & Huber, 2002; Poulet & Hedwig, 2006; Schöneich & Hedwig, 2010). Male crickets typically produce pure-tone calling songs with dominant frequencies ranging from approximately 2 to 8 kHz, around 5 kHz in most field crickets (Michelsen, 1998). These calls are used to attract females and are generated by stereotyped opening and closing movements of the forewings. Sound production relies on two successive processes, stridulation and resonant amplification (Michelsen, 1998), which together define the basic call unit, or syllable, corresponding to a single closing movement of the forewings.

**Figure 1:**
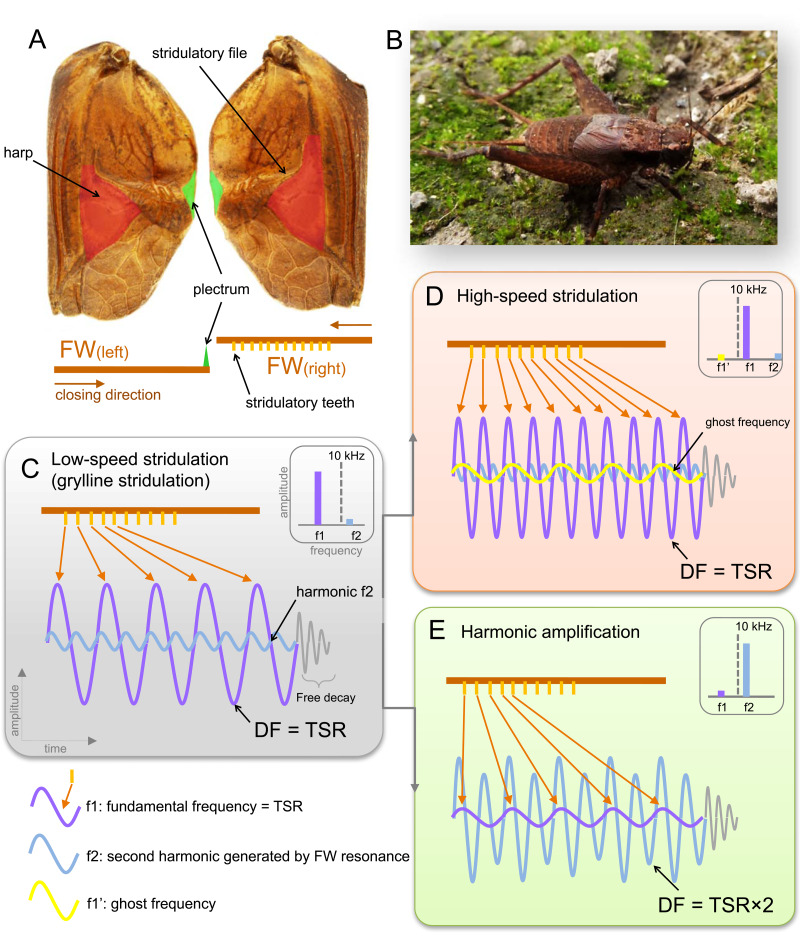
Sound production mechanism in *Agnothecous*. (A) Photographs of the left and right forewings of *Agnothecous robustus* (up) with a schematic representation of the closing forewing movement during the stridulation (down). (B) Photograph of a male individual of the species *Agnothecous sarramea*, dorsal view, in life. (C–E) Relation between the tooth-strike rate and the frequency components of the call in the classic, low-frequency sound production mechanism in grylline species (C), in the two high-frequency sound production mechanisms in *Agnothecous* (D, E). DF, dominant frequency; TSR, tooth strike rate.

During stridulation, a plectrum on the left forewing rubs against a stridulatory file, a series of cuticular teeth on the right forewing, producing a series of mechanical pulses, the “vibratory signal” (Fig. 1A). The main frequency component of this signal, the carrier frequency, is determined by the tooth strike rate, which depends on both behavioural parameters (such as wing-closing speed) and morphological parameters (such as tooth density). The vibratory signal is then transmitted to membranous regions of the forewings acting as resonators, most notably the harp, which amplifies and filters the sound (Bennet-Clark, 1970; Nocke, 1971; Michelsen & Nocke, 1974; Bennet-Clark, 1999; Bennet-Clark, 2003; Montealegre-Z, Jonsson & Robert, 2011). In most crickets, the dominant frequency of the emitted call corresponds to the carrier frequency of the vibratory signal, resulting in highly tonal calls.

However, most species belonging to the tribe Lebinthini (subfamily Eneopterinae) depart markedly from this pattern by producing calls with much higher dominant frequencies, ranging from approximately 10 to 28 kHz (*e.g.*, Robillard & Desutter-Grandcolas, 2004; Tan et al., 2021). In these species, high-frequency calling has been interpreted as part of a derived system of intersexual communication (ter Hofstede et al., 2015). Notably, many Lebinthini species produce harmonic-dominant calls, whereby the dominant frequency does not correspond to the fundamental frequency, but to a higher harmonic of the spectrum (Robillard, Grandcolas & Desutter-Grandcolas, 2007; Tan et al., 2021).

The New Caledonian genus *Agnothecous* Saussure, 1878 exemplifies this diversity. Species within this genus are relatively conserved in external morphology, yet produce calls with dominant frequencies spanning roughly 10 to 20 kHz. A previous functional study on *Agnothecous robustus* (Chopard, 1915) demonstrated that its high-frequency call is generated through accelerated stridulatory movements, representing a primarily behavioural modification of sound production (Robillard et al., 2013). In this species, the fundamental frequency constitutes the dominant spectral peak, while a lower-frequency component persists as a relictual signal, termed a “ghost frequency” (Robillard et al., 2013). Subsequent observations across the Lebinthini tribe indicate that this mechanism is not universal. In several taxa, high-frequency calls appear instead to result from changes in the resonant properties of the forewings membrane, leading to amplification of harmonic vibrations. This alternative pathway is driven primarily by modifications of morphological traits affecting the physical properties of the resonator (*e.g.*, *Gnominthus baitabagus* Vicente & Robillard, 2015: Vicente et al., 2015; *Ligypterus* Saussure, 1878: Robillard et al., 2015; *Ponca hebardi* Robillard, 2005: Gaiddon et al., 2026).

These observations suggest that similar acoustic phenotypes, *i.e.,* high-frequency calling, may be achieved through distinct evolutionary routes, involving either behavioural changes, morphological changes, or their combination. Closely related species may therefore differ not only in their acoustic signals, but also in the underlying mechanisms producing them. This raises the broader question of how behavioural and morphological traits interact during acoustic signal diversification, and whether particular evolutionary strategies are conserved or repeatedly reinvented.

In this study, we address this question using a comparative and phylogenetic analysis of the genus *Agnothecous*. Our objectives are threefold. First, we identify the sound-production mechanisms underlying high-frequency calls in 15 *Agnothecous* species, testing whether dominant frequencies are produced by *high-speed stridulation* or by *harmonic amplification* through the harp. Second, we assess the evolutionary history of these mechanisms to determine whether they are conserved across the genus or have evolved independently, and to test whether ghost frequencies represent an ancestral condition or multiple convergent outcomes. Third, we quantify the joint evolution of key morphological and behavioural parameters to evaluate how their interplay has shaped the diversification of the acoustic signals in *Agnothecous*. By explicitly distinguishing behavioural and morphological axes of variation, this study provides a mechanistic and evolutionary framework for understanding how complex acoustic signals diversify in closely related species.

## Materials and Methods

### Taxonomic sampling and study material

The genus *Agnothecous* comprises 22 described species endemic to New Caledonia (Robillard, Nattier & Desutter-Grandcolas, 2010; Cigliano et al., 2025; Le Flanchec et al., 2025). We analysed 15 species (Table 1), representing the majority of the known diversity of the genus at the time of the study. Specimens were collected in Province Nord and Province Sud, New Caledonia, in accordance with local regulations. Direction du Développement Économique et de l’Environnement, Province Nord and Province Sud provided the permit for field work in New Caledonia. Acoustic recordings were conducted in natural habitats, primarily in forested areas distant from human disturbance. Seven species were not included in the analyses. Four species lacked available acoustic data (*A. humboldti* Robillard, 2010; *A. nekando* Robillard, 2010; *A. novaecaledoniae* Gorochov, 1986; *A. petchekara* Desutter-Grandcolas, 2010), and three species (*A. anonymous* Le Flanchec, Vendanger & Robillard, 2025; *A. borendyi* Le Flanchec, Vendanger & Robillard, 2025; *A. kwakwe* Le Flanchec, Vendanger & Robillard, 2025) were described after the completion of data collection.

### Acoustic recordings and measurements

#### Recordings

Calls were recorded both in the field and in laboratory conditions using a modified condenser microphone capsule CM16 (Avisoft Bioacoustics, Berlin, Germany; frequency range 3–150 kHz ± 6 dB; R. Specht, personal communication). Field recordings were made with a Tascam HD-P2 digital recorder (96 kHz sampling rate, 16 bit), whereas laboratory recordings used Avisoft Triggering Harddisk Recorder (v.2.97) coupled with a 8pre MOTU sound card at the same sampling rate.

In total, 93 male specimens from 15 species were recorded (mean = 6.2 males per species; Table 1). For each individual, a call bout containing up to 20 calls (mean = 11) was selected for acoustic analyses. Voucher recordings were deposited on xeno-canto (see Supplemental Files) and in the Sound Library of the Muséum national d’Histoire naturelle (MNHN; Table S1).

**Table 1:** Taxonomic and acoustic sampling of the 15 *Agnothecous* species analysed.

	Number of recorded males	Number of calls (mean per male)	Number of males for morphological study
*A. albifrons* Desutter-Grandcolas, 1997	7	57 (8)	5
*A. azurensis* Desutter-Grandcolas, 2006	17	251 (15)	10
*A. brachypterus* Gorochov, 1986	10	94 (9)	5
*A. chopardi* Desutter-Grandcolas, 2006	1	8	1
*A. clarus* Desutter-Grandcolas, 2006	1	11	1
*A. doensis* Desutter-Grandcolas, 2006	1	9	5
*A. meridionalis* Desutter-Grandcolas, 2006	11	127 (12)	5
*A. minoris* Robillard, 2010	1	20	1
*A. obscurus* (Chopard, 1970)	11	172 (16)	4
*A. occidentalis* Desutter-Grandcolas, 2006	6	80 (13)	5
*A. pinsula* Robillard, 2010	3	16 (5)	3
*A. robustus* (Chopard, 1915)	5	60 (12)	4
*A. sarramea* Desutter-Grandcolas, 1997	3	23 (8)	5
*A. tapinopus* Saussure, 1878	12	118 (10)	4
*A. yahoue* Otte, 1987	4	20 (5)	5
**TOTAL**	**93**	**1,066 (11)**	**63**

#### Acoustic traits

We focused on three acoustic traits involved in sound production: dominant frequency, syllable duration, and syllable amplitude profile. Measurements were performed using Avisoft-SASLab Pro v.4.40 (Specht, 2009) with the automated “pulse train analysis” tool. In several species, the syllables contained short silent intervals resulting from discontinuous forewing closure (Robillard et al., 2013). To account for these pauses, we estimated a corrected syllable duration representing the effective duration of sound production during active wing closure. This correction was performed on a subsample of 1–5 recordings per species by manually removing silent intervals from 10 syllables per recording (Fig. S2). Corrected syllable duration was subsequently used to estimate behavioural parameters related to stridulation (see below).

### Morphological measurements

#### Stridulatory file

Morphological analyses were conducted on male specimens held in the MNHN collections. One to five males per species were examined (mean = 4; total = 62 forewings), prioritising acoustically recorded individuals when available. Right forewings were detached, softened with cotton soaked in water, cut at its basis, and mounted for imaging.

Stridulatory files were imaged using a scanning electron microscope (SEM) from the “Plateforme de Microscopie Électronique” of the MNHN. Pictures obtained with magnification ×700 were necessary for accurate measurements of inter-tooth distances. Given the dimensions of the files, several photos per file were necessary and were combined using the Photomerge function of the program Adobe Photoshop CS6. File measurements were obtained on SEM images (Fig. S1) using the dimension tool of the program ImageJ v.1.45s (Schneider, Rasband & Eliceiri, 2012). Inter-tooth distances for each pair of teeth were measured as the distance from the apex of one tooth to the apex of the next one (Data S1). To determine the functional part of the stridulatory file (*i.e.,* the part swept by the plectrum during stridulation), inter-tooth distances were plotted against their corresponding tooth number (Fig. S1). The functional region of the file was identified following established criteria based on the progressive increase in inter-tooth distances from the anal to the basal part (Gu et al., 2012). From this region, we measured length of file functional part, number of functional teeth, and mean inter-tooth distance in the functional region.

#### Harp morphology

The harp, identified as the main resonating area of the forewing (*e.g.*, Montealegre-Z, Jonsson & Robert, 2011; Robillard et al., 2013), was photographed using a digital microscope camera (AmScope MU1000, ×20 magnification; http://www.Amscope.com). Harp surface area was measured in ImageJ.

### Behavioural trait estimation

Behavioural parameters were estimated by combining acoustic and morphological measurements.

• Global stridulation speed was calculated as the length of the functional region of the stridulatory file divided by the corrected syllable duration.
• Tooth strike rate was calculated as the number of functional teeth divided by the corrected syllable duration.
• Estimated instantaneous speed, defined as the mean velocity of the forewings between two successive stridulatory teeth, was calculated as the product of mean inter-tooth distance and dominant frequency.
• Sound ratio was calculated as the ratio between corrected syllable duration and raw syllable duration, quantifying the proportion of active sound production within each syllable.

### Determination of sound-production mechanism

The sound-production mechanism of each species was inferred by comparing tooth strike rate, dominant frequency, and stridulation speed estimates. Two complementary indirect criteria were applied to all species:

• Comparison between estimated tooth strike rate and dominant frequency. When the dominant frequency matches the tooth strike rate, sound is produced following the usual *grylline* mechanism, in which each tooth strike produces one oscillation of the sound wave. In contrast, if the dominant frequency is substantially higher, particularly when it corresponds to an integer multiple of the tooth strike rate, the dominant frequency corresponds to a harmonic of the fundamental frequency.
• Comparison between global stridulation speed and estimated instantaneous speed. The estimated instantaneous speed assumes a 1:1 ratio between tooth strikes and sound oscillations (*grylline* mechanism). If this assumption holds, global stridulation speed will align with the estimated instantaneous speed. By contrast, when these two values diverge, specifically if the instantaneous speed is much higher, it indicates that the sound corresponds to a harmonic, as the tooth strikes are too slow to produce that specific dominant frequency.

In addition, the call of *A. obscurus* (Chopard, 1970) was recorded using a high-speed video camera (3,000 frames s^−1^; AOS Technologies AG, Baden-Daettwil, Switzerland) for direct measurements of forewing kinematics. Additional data were also available for the species *A. robustus* using previously published laser displacement data (Robillard et al., 2013). These data were used to validate the indirect criteria.

Each species was assigned to one of two sound-production mechanisms, coded as a binary trait for comparative analyses. Full methodological validation and direct kinematic measurements are described in the Results and in the Supplementary Materials.

### Phylogenetic reconstruction

#### Molecular data

We revisited the phylogeny of the genus using the same 15 described species, based on the study of Nattier et al. (2012). We used four species as outgroups for the phylogenetic reconstruction: one other species of Lebinthini, *Microbinthus santoensis* (Robillard, 2009), two representing two other tribes of Eneopterinae, *Nisitrus malaya* Robillard & Tan, 2021 and *Eneoptera guyanensis* Chopard, 1931, and one species belonging to the subfamily Gryllinae, *Acheta domesticus* (Linnaeus, 1758) (Table S2).

We used the molecular data previously published in Nattier et al. (2012), consisting of 10 molecular markers (ca. 6.8 kb). We concatenated the mitochondrial (five markers) and nuclear data (five markers) using the software Sequence Matrix v.1.7.7 (Vaidya, Lohman & Meier, 2011). The concatenated dataset was then pruned to keep one specimen per species to reconstruct the species tree and run dating analyses.

#### Phylogenetic inference

We applied the Bayesian relaxed uncorrelated log-normal approach implemented in BEAST v1.8.1 (Suchard et al., 2018) to estimate divergence ages of the lineages. The calibration analysis was set following Nattier et al. (2012) using a normal distribution for the tree prior to calibrate node with a standard deviation of 1%, and an age of 20.2 Myr for *Agnothecous* (with 95% highest posterior density interval of 14.9–25.82) obtained from Anso et al. (2016). We ran four Markov chains simultaneously for 15 million generations, sampling every 1,000 generations to ensure the independence of samples. After checking for convergence, a 10% burn-in was applied, and a majority rule consensus tree was generated using Tree Annotator, which retained the Maximum Clade Credibility (MCC) tree with branch length equal to the median lengths of all the trees.

### Comparative and evolutionary analyses

#### Ancestral state reconstruction of sound-production mechanisms

We first reconstructed the ancestral states of the binary trait corresponding to the type of sound-production mechanism across 15 *Agnothecous* species and four outgroups. To account for phylogenetic uncertainty, we randomly sampled 100 trees from the posterior distribution obtained from the BEAST analysis. Ancestral state reconstructions were performed using the Multistate model in BayesTraits v3.0.2 (Pagel, Meade & Barker, 2004; Meade & Pagel, 2022), as implemented in RASP v4 (Yu, Blair & He, 2020).

For each tree, Markov Chain Monte Carlo (MCMC) analyses were run for 1,000,000 iterations, with a burn-in of 10,000. The marginal likelihoods of alternative models were estimated using the stepping-stone method (“stones” function) to compute Bayes Factors (BF) and assess support for particular evolutionary scenarios.

#### Correlated evolution of continuous traits

To explore evolutionary correlations among the 11 continuous traits related to morphology, behaviour, and acoustic signals (see below and Table 2), we used BayesTraits’ continuous trait correlation framework. Pairwise correlations between traits were tested by comparing the marginal likelihoods of two models: one assuming a correlation of zero (null model), and one allowing the traits to covary (alternative model). Bayes Factors were calculated to evaluate model support.

**Table 2:** Traits involved in sound production and inferred mechanism for each species in the study. Data for *A. robustus* are extracted from Robillard et al. (2013). Estimated instantaneous speed is given only for purpose of comparison with the global speed variable to determine the type of sound production mechanism but was not considered in the comparative analyses. The type of sound production mechanism was determined by comparing the matches and mismatches between dominant frequency and tooth strike rate, and between global speed and estimated instantaneous speed (see ‘Material and methods’ for details). Species shown in bold correspond to those exhibiting high-frequency stridulation.

**Species**	**Morphological traits**					**Acoustic traits**			**Behavioural traits**				**Type of sound production mechanism**
	Body size (Pronotum length, mm)	Harp surface (mm^2^)	Number of functional teeth	Inter-tooth distance (μm)	File length (mm)	Dominant frequency (kHz)	Syllable duration (ms)	Corrected syllable duration (ms)	Sound ratio	Tooth strike rate (teeth s^−1^)	Global speed (mm s^−1^)	Estimated instantaneous speed (mm s^−1^)	
*A. albifrons*	3.52	1.71	53	13.89	0.74	16.28	20.05	10.41	0.52	5,089	71.06	226	Harmonic amplification
*A. azurensis*	3.25	1.56	76.5	10.71	0.82	15.65	15.03	11.03	0.73	6,979	74.33	168	Harmonic amplification
*A. brachypterus*	3.20	1.56	90	11.12	1.00	18.08	15.96	11.54	0.72	7,797	86.64	201	Harmonic amplification
*A. chopardi*	3.00	1.32	68	11.01	0.75	16.94	12.69	10.01	0.80	6,793	74.92	187	Harmonic amplification
*A. clarus*	2.80	1.31	52	11.41	0.59	18.61	17.93	11.26	0.63	4,619	52.41	212	Harmonic amplification
*A. doensis*	3.61	1.62	79	10.49	0.83	13.93	11.78	11.78	1.00	6,705	70.36	146	Harmonic amplification
*A. meridionalis*	3.70	1.34	74	11.87	0.88	15.16	19.92	11.89	0.60	6,226	74.04	180	Harmonic amplification
*A. minoris*	2.80	1.70	80	12.28	0.98	13.52	12.46	12.46	1.00	6,259	76.68	166	Harmonic amplification
*A. obscurus*	3.70	1.88	73	14.04	1.02	15.07	19.32	11.28	0.58	6,473	90.44	212	Harmonic amplification
*A. occidentalis*	3.80	1.77	78	11.68	0.92	14.91	10.67	9.07	0.85	8,598	101.41	174	Harmonic amplification
*A. pinsula*	3.06	1.26	77	10.64	0.85	18.45	18.11	10.45	0.58	7,371	81.37	196	Harmonic amplification
***A. robustus***	**4.59**	**2.78**	**95**	**15.6**	**1.48**	**10.89**	**9.91**	**8.79**	**0.89**	**10,811**	**168.42**	**170**	**High-frequency stridulation**
*A. sarramea*	3.91	1.62	76	11.21	0.85	13.10	10.38	11.26	1	6,750	75.50	147	Harmonic amplification
***A. tapinopus***	**4.25**	**2.22**	**64**	**13.59**	**0.87**	**11.20**	**8.56**	**6.89**	**0.80**	**9,288**	**126.26**	**152**	**High-frequency stridulation**
*A. yahoue*	3.25	1.47	73	12.26	0.89	16.28	14.83	11.85	0.80	6,160	75.11	200	Harmonic amplification

For all analyses, MCMC performance and parameter convergence were assessed using Tracer v1.7.1 (Rambaut et al., 2018). Only runs with effective sample sizes >200 and posterior distributions with narrow credibility intervals were retained.

#### Evolutionary models and ancestral state reconstruction of continuous traits

To infer the evolutionary history of the 11 continuous traits of the 15 *Agnothecous* species, we carried out ancestral state reconstructions using a time-calibrated phylogeny and comparative methods implemented in R (*v.* 4.3.1; R Core Team, 2023). We imported our time-calibrated phylogeny of *Agnothecous* using the package *ape* (Paradis, Claude & Strimmer, 2004), and outgroup taxa were pruned with *keep.tip()* to retain only *Agnothecous* species. The resulting pruned tree was used for all downstream analyses.

The studied traits include (see Table 2):

• Morphological traits: body size, harp surface, file length, inter-tooth distance, and number of functional teeth.
• Behavioural traits: global speed, tooth strike rate, and sound ratio.
• Acoustic traits: syllable duration, corrected syllable duration, and dominant frequency.

Prior to analyses, all continuous variables were log-transformed to improve normality and reduce heteroscedasticity. For each trait, we compared three models of continuous trait evolution: Brownian Motion (BM), Ornstein–Uhlenbeck (OU), and Early Burst (EB), using the *fitContinuous()* function from the package *geiger* (Harmon et al., 2008). Model fits were compared using Akaike Information Criterion (AIC), and Akaike weights (AICw) were computed to evaluate relative support for each model. We reconstructed ancestral trait values at internal nodes using the *fastAnc()* function from *phytools* (Revell, 2012), assuming a BM model, except for dominant frequency, for which a OU model was better supported. For dominant frequency, we then used the package *OUwie* (Beaulieu et al., 2012). Ancestral state reconstructions were visualised using *contMap()* from the package *phytools*, which produces color-gradient trees indicating the inferred trait values along each branch. Confidence intervals at nodes were retrieved from *fastAnc()* outputs and plotted where relevant.

## Results

### Phylogenetic framework and trait dataset

**Figure 2:**
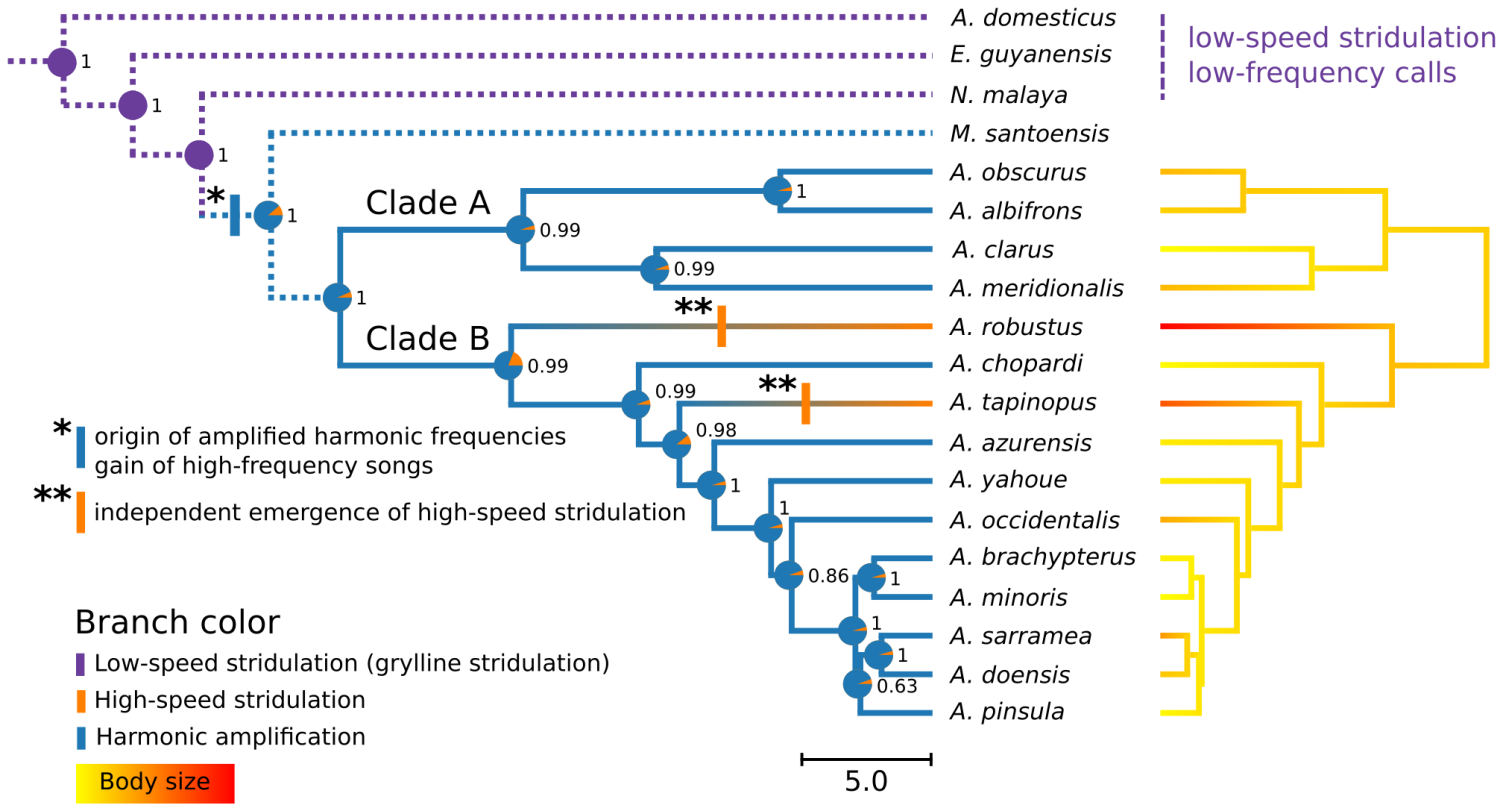
Reconstructed phylogeny of 15 species of the genus *Agnothecous*, and ancestral states reconstruction for the type of sound production mechanism based on the 2 models of high-frequency sound production. Reconstructed evolutionary origins of the *harmonic amplification* and *high-speed stridulation* models are indicated with asterisks. Branch lengths for solid branches in the phylogeny are indicated using the scale below the tree. Deeper branches and nodes in the Gryllidae phylogeny indicated with dotted lines with branch lengths not corresponding to reality. Support values for each reconstructed node are indicated. The reconstructed phylogeny with ancestral states reconstruction for the average body size (right) is shown for comparison purposes.

The phylogenetic relationships among the 15 *Agnothecous* species are shown in Fig. 2. The inferred topology is congruent with previous phylogenetic studies (Nattier et al., 2012; Le Flanchec et al., 2025), recovering two main clades (clade A and clade B) of comparable age and generally strong node support. Across the genus, we quantified 11 traits related to morphology, behaviour, and acoustics (Table 2). These traits exhibit contrasting levels of interspecific variation, providing a suitable framework to investigate both acoustic diversity and underlying sound-production mechanisms.

### Acoustic, morphological and behavioural diversity

#### Acoustic traits

Syllable duration varies markedly among species, ranging from 8.6 ms in *A. tapinopus* Saussure, 1878 to 20.1 ms in *A. albifrons* Desutter-Grandcolas, 1997*.* In contrast, corrected syllable duration shows limited variation across most species, typically ranging between 10 and 12 ms, with notable exceptions in *A. occidentalis* Desutter-Grandcolas, 2006 (9.07 ms), *A. robustus* (8.79 ms), and *A. tapinopus* (6.89 ms).

Dominant frequency exhibits substantial interspecific variation, spanning from 10.9 kHz in *A. robustus* to 18.6 kHz in *A. clarus* Desutter-Grandcolas, 2006, indicating pronounced divergence in spectral properties despite relatively conserved syllable structure.

#### Morphological traits

Stridulatory files show the typical cricket pattern of progressively increasing inter-tooth distances from the anal to the basal region (Fig. S1). Harp surface area varies substantially among species, from 1.26 mm^2^ in *A. pinsula* Robillard, 2010 to 2.78 mm^2^ in *A. robustus*. The number of functional teeth ranges from 52 (*A. clarus)* to 95 (*A. robustus)*, with file lengths spanning from 0.59 mm (*A. clarus*) to 1.48 mm (*A. robustus*). Mean inter-tooth distances range from 10.49 μm (*A. doensis* Desutter-Grandcolas, 2006) to 15.6 μm (*A. robustus*), highlighting significant morphological variation in the stridulatory apparatus.

#### Behavioural traits

Behavioural parameters also show strong interspecific divergence. *A. robustus* stands out as the most extreme species, displaying both the highest global stridulation speed (168 mm s^−1^) and the highest tooth strike rate (10,811 teeth s^−1^).

The closing pattern of the forewings, quantified by the sound ratio, differs markedly among species. Eight species (*A. chopardi* Desutter-Grandcolas, 2006*; A. doensis; A. minoris* Robillard, 2010*; A. occidentalis; A. robustus; A. sarramea* Desutter-Grandcolas, 1997; *A. tapinopus*; and *A. yahoue* Otte, 1987) exhibit quasi-continuous wing closure, with syllables containing less than 20% silence (*i.e.,* sound ratio ≥ 80), whereas the remaining species show discontinuous closure patterns, with silent intervals accounting for 27% (*A. azurensis* Desutter-Grandcolas, 2006) to 48% (*A. albifrons*) of syllable duration.

### Identification of sound-production mechanisms

Together, indirect estimates and direct kinematic measurements consistently support the existence of two sound-production mechanisms within *Agnothecous*. In most species, the dominant frequency in the call corresponds to the carrier frequency of the vibratory signal of the stridulation, which is directly generated by the tooth strike rate. Under this first mechanism, named high-speed stridulation (Fig. 1D), the species stridulation follows the usual grylline mechanism of stridulation (*i.e.,* dominant frequency ≈ tooth strike rate) (*e.g.*, Montealegre-Z, 2009). High-frequency calls under this mechanism are produced by the means of an increased tooth strike rate. In some species, the lowest, non-dominant frequency component in the spectrum, may correspond to a relictual frequency named “ghost frequency” (Robillard et al., 2013), a vestigial trace of an ancient fundamental frequency, while the tooth strike rate generates the higher carrier frequency corresponding to the dominant frequency of the call, resembling, misleadingly, to an amplified harmonic of the lowest peak.

In other species, the dominant frequency of the call corresponds to a harmonic frequency of the fundamental, *i.e.,* a multiple integer of the carrier frequency produced by the stridulation. Under this alternative mechanism, named *harmonic amplification* (Fig. 1E), the stridulation produces a low-frequency tooth strike rate corresponding to the fundamental frequency of the call spectrum, but the dominant frequency corresponds to a harmonic frequency of the fundamental amplified through the resonance of the forewings (*i.e.,* dominant frequency ≈ n×tooth strike rate, where n is the harmonic number in the call spectrum). High-frequency calls under this mechanism are produced by exploiting resonant properties of the forewings different from those of grylline species, allowing the amplification of high-frequency components of the vibratory signal of the stridulation.

#### Comparison of tooth strike rate and dominant frequency

Comparison between tooth strike rate (teeth s^−1^) and dominant frequency (kHz) reveal two distinct patterns (Table 2). In *A. robustus* and *A. tapinopus*, tooth strike rate closely matches dominant frequency (1 teeth s^−1^ for 1 Hz), indicating that the dominant spectral component corresponds to the carrier frequency generated directly by stridulation.

In all other species, dominant frequency is substantially higher than tooth strike rate. In *A. doensis* and *A. minoris*, dominant frequency is approximately twice the tooth strike rate, whereas in *A. albifrons* and *A. clarus,* it reaches approximately three- to fourfold higher values. These discrepancies indicate that the dominant frequency does not correspond to the carrier frequency generated by tooth strikes.

#### Comparison of global and instantaneous stridulation speed

The same dichotomy emerges from comparisons between global stridulation speed and estimated instantaneous speed (Table 2). In *A. robustus* and *A. tapinopus*, global speed closely approximates instantaneous speed (global speed = 168 mm s^−1^, estimated instantaneous speed = 170 mm s^−1^ for *A. robustus*; global speed = 126 mm s^−1^, estimated instantaneous speed = 152 mm s^−1^ for *A. tapinopus*), consistent with direct generation of the dominant frequency through stridulation.

In all other species, global speed is markedly lower than estimated instantaneous speed based on dominant frequency, further supporting the conclusion that dominant frequency corresponds to an amplified harmonic rather than to the fundamental stridulatory frequency.

#### Validation using high-speed video

High-speed video recordings of *A. obscurus* (Video S1) provide direct evidence supporting the *harmonic amplification* mechanism. The measured forewing closing speed (61 ± 11 mm s^−1^) is slightly lower than the global speed trait value for this species (90 mm s^−1^). Based on this closing speed and the number of functional teeth, the expected dominant frequency generated by tooth strikes alone is approximately 4.5 kHz, whereas the dominant frequency actually observed in the call spectrum reaches ∼15 kHz. This marked discrepancy demonstrates that the dominant frequency cannot be produced directly by stridulation and rather corresponds to an amplified harmonic component. Video recordings further confirm the discontinuous forewing closure pattern inferred from acoustic analyses (Video S1). By comparison, the forewing closing speed of *A. robustus* has been estimated at 168 mm s^−1^ by Robillard et al. (2013), this high velocity allowing this species to generate a high tooth strike rate.

### Evolutionary history of sound-production mechanisms

Ancestral state reconstruction results indicate that the most recent common ancestor of *Agnothecous* likely produced calls dominated by a harmonic frequency (Fig. 2). The *harmonic amplification* mechanism appears to have originated early within the Lebinthini lineage, and is retained in most extant *Agnothecous* species.

In contrast, the *high-speed stridulation* mechanism evolved independently at least twice within the genus, in *A. robustus* and *A. tapinopus* (Fig. 2). These shifts are associated with marked increases in stridulation speed and tooth strike rate, and with the re-emergence of a dominant fundamental frequency.

### Correlated evolution of morphological, behavioural and acoustic traits

**Figure 3:**
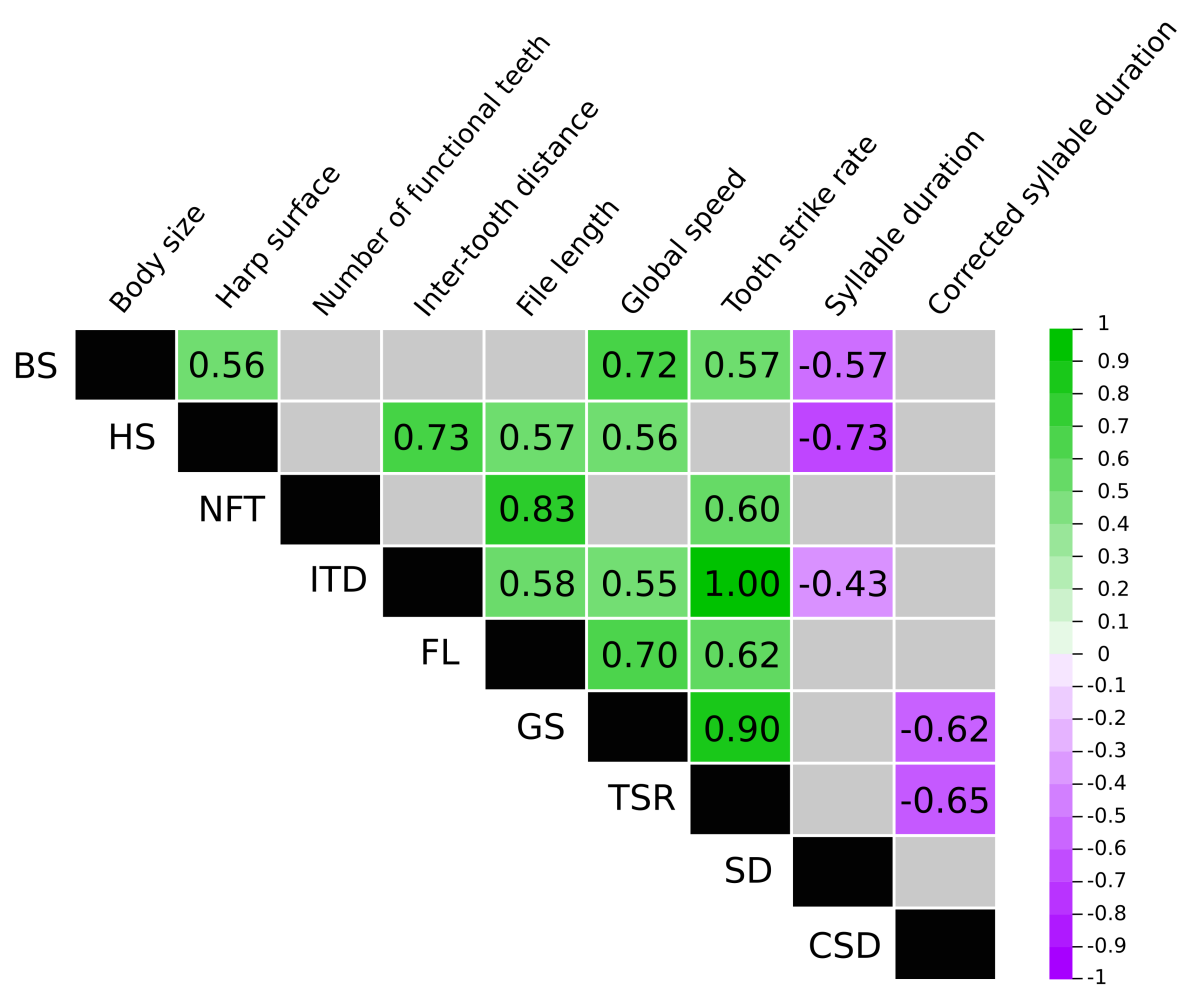
Correlation table for 9 continuous traits describing the sound production mechanism of *Agnothecous* species. Dominant frequency and sound ratio have been excluded from the table, as no significant correlation could be found between any pairs containing these traits. Correlation is characterised for each pair of traits by *R*^2^, represented by a colour scale (green indicate positive correlation, purple indicate negative correlation). Grey cases show pairs where no significant correlation could be found.

Bayesian correlation analyses reveal extensive covariation among morphological and behavioural traits (Fig. 3). Body size shows positive correlations with harp surface, global stridulation speed, and tooth strike rate, and a negative correlation with syllable duration.

Harp surface correlates positively with inter-tooth distance, file length, and global speed, and negatively with syllable duration. In contrast, the number of functional teeth shows no correlation with body size of harp surface, but correlates positively with tooth strike rate.

Inter-tooth distance, file length, global speed, and tooth strike rate form a tightly correlated trait cluster, while syllable duration and corrected syllable duration are negatively correlated with stridulation speed parameters. These results highlight a strong coupling between morphological dimensions of the stridulatory apparatus and behavioural parameters of wing movement.

### Evolution of continuous traits

**Figure 4:**
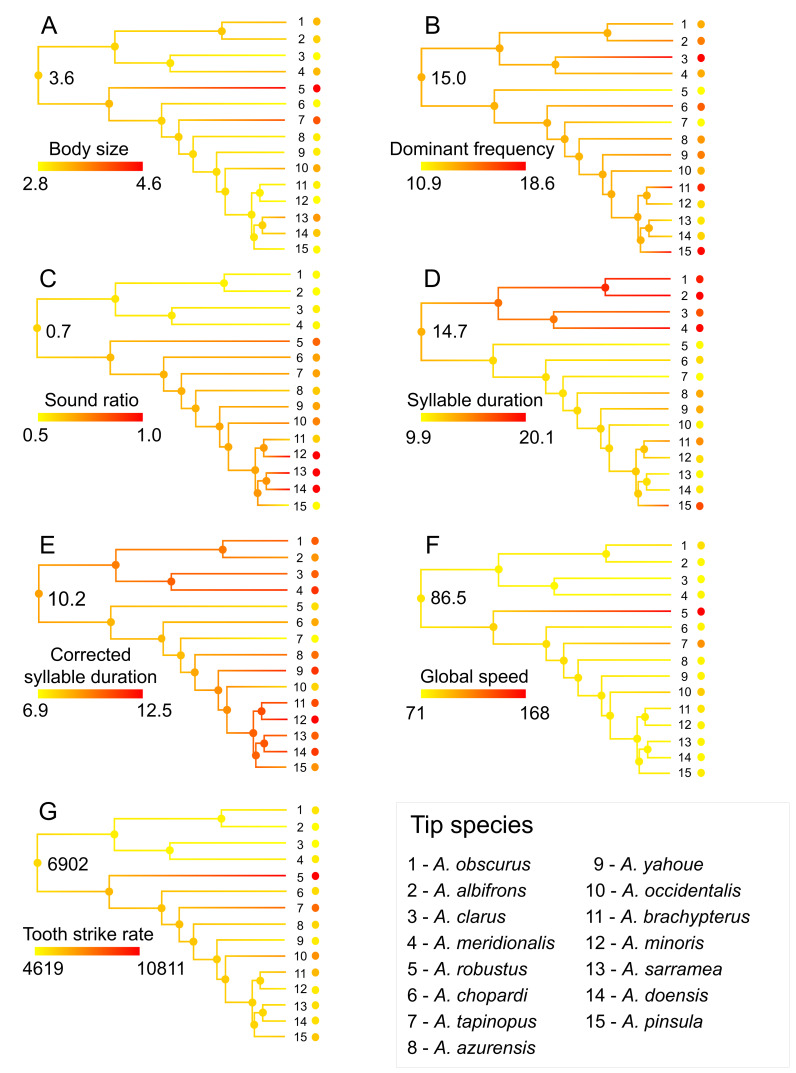
Ancestral state reconstructions for seven continuous traits analysed in the 15 sampled species in *Agnothecous*. Variation of each trait value along phylogeny branches is indicated with a colour scale. Traits values for the 15 tip species are indicated with a coloured circle. Reconstructed ancestral values at the base of each phylogeny is indicated.

Ancestral state reconstructions of key traits are shown in Fig. 4 (additional traits in Fig. S3). Body size and harp surface show moderate ancestral values, with independent increases in *A. robustus* and *A. tapinopus* (Fig. 4A). File length and inter-tooth distance broadly track body size, with notable deviations in several species.

Dominant frequency shows weak phylogenetic signal and predominantly evolves through changes at terminal branches (Fig. 4B). Decreases in dominant frequency are associated with the independent evolution of *high-speed stridulation* in *A. robustus* and *A. tapinopus*, whereas the highest dominant frequencies arise independently in several small-bodied species using *harmonic amplification*.

Sound ratio and syllable duration show strong clade-level structuring (Figs. 4C–4D). Species in clade A tend to exhibit discontinuous wing closure and longer syllables, whereas species in clade B show more continuous closure patterns and shorter syllables. Corrected syllable duration lacks this clade structure (Fig. 4E), confirming that variation in syllable duration largely reflects differences in silent intervals rather than in active sound production.

Global stridulation speed and tooth strike rate show pronounced increases only in *A. robustus* and *A. tapinopus*, consistent with their shift toward *high-speed stridulation* (Figs. 4F–4G).

## Discussion

### Multiple sound-production mechanisms in closely related species

Sound production in insects is often described as stereotyped and mechanically simple compared to vertebrates (*e.g.*, Gerhardt & Huber, 2002; Hedwig, 2014). In ensiferans and cicadas, acoustic diversity is usually attributed to variation in temporal patterns built from stereotyped sound units (*e.g.*, Alexander, 1962; Otte, 1992; Fonseca & Hedwig, 2014). However, this view underestimates the diversity of both sound units and frequency spectra, even among closely related species. Our results in *Agnothecous* show that evolutionary changes in sound production involve not only temporal organisation, but also distinct mechanical and behavioural pathways.

In most crickets, low-frequency calls are produced according to the *grylline stridulation* mechanism (Fig. 1C), in which the carrier frequency (determined by the tooth strike rate) matches the dominant fundamental frequency of the emitted sound unit (*e.g.*, Michelsen, 1998; Bennet-Clark, 2003; Montealegre-Z, Jonsson & Robert, 2011). By contrast, most Lebinthini (*e.g.*, Robillard & Desutter-Grandcolas, 2004; Robillard & Desutter-Grandcolas, 2011; Tan et al., 2021), including all but two *Agnothecous* species, use the *harmonic amplification* mechanism (Fig. 1E). In this system, the dominant frequency corresponds to an integer multiple (typically the second harmonic) of the fundamental frequency, and by extension, of the carrier frequency generated by the stridulation.

A central result of this study is that high dominant frequencies in *Agnothecous* calls arise through two distinct mechanisms. Phylogenetic reconstructions indicate that the most recent common ancestor of *Agnothecous* likely used the *harmonic amplification* mechanism, inherited from Lebinthini ancestors and characterised by second-harmonic dominance (Tan et al., 2021; Le Flanchec et al., 2025). Consistent with this scenario, most species in the genus produce a low carrier frequency through slow stridulation (low tooth strike rate), while forewing resonators preferentially amplify the second harmonic.

By contrast, *A. tapinopus* and *A. robustus* generate high-frequency calls through a different mechanism: *high-speed stridulation.* In these species, increased tooth strike rate directly set the dominant frequency, making their sound production functionally equivalent to the *grylline stridulation* mechanism. This confirms and extends earlier observations for *A. robustus* (Robillard et al., 2013).

It is important to note that this shift does not result from morphological changes in the stridulatory file. Both species exhibit low tooth densities (*A. robustus*: ∼64.1 teeth mm^−1^; *A. tapinopus*: ∼73.6 teeth mm^−1^; Table 2), among the lowest values observed in the genus. Instead, high tooth strike rates are achieved behaviourally, through markedly increased forewing closure speeds (168 mm s^−1^ in *A. robustus*; 126 mm s^−1^ in *A. tapinopus*; Table 2). This behavioural acceleration produces very short syllables (8.6 ms in *A. tapinopus*; 9.9 ms in *A. robustus*) with high sound ratios (∼0.9 in *A. robustus*; 0.8 in *A. tapinopus*), reflecting nearly continuous wing motion. Together, these results demonstrate that behavioural change (increase in the speed of the forewings movement) rather than morphological modification (no increase in tooth density), underlies the emergence of *high-speed stridulation* in these species.

Despite their distinct mechanical bases, the two mechanisms produce remarkably similar acoustic outputs. In both cases, the call spectrum shows a dominant high-frequency peak, the second harmonic of a weakly amplified low frequency. However, the nature of this low-frequency peak differs. Under *harmonic amplification*, it corresponds to the true fundamental frequency. Under *high-speed stridulation*, it likely represents a relictual fundamental frequency, called “ghost frequency” (Robillard et al., 2013).

Ancestral state reconstructions indicate that *high-speed stridulation* evolved convergently in *A. robustus* and *A. tapinopus* from an ancestral *harmonic amplification* mechanism. Functionally, *high-speed stridulation* is a “high-speed” version of the *grylline stridulation* mechanism. Mechanistically, however, the transition occurred from a system already specialised for harmonic dominance. This distinction is important, as it suggests that traces of the ancestral system, such as ghost frequencies, may persist. More broadly, this case illustrates how different evolutionary and mechanical pathways can lead to similar acoustic signals, highlighting the plasticity of cricket sound production and the central role of behavioural modulation.

### Compensation strategies and irreversibility of high-frequency communication

Both species that convergently evolved *high-speed stridulation* are also the largest in the genus. Ancestral state reconstructions show that increases in body size occurred independently in *A. tapinopus* and *A. robustus*, paralleling changes in sound production mechanisms (Fig. 4A). Although our sample size limits formal tests, it is plausible that only large-bodied males can sustain the muscular power required for the high tooth strike rates associated with this mechanism. Producing such high tooth strike rates likely requires elevated muscular power and contraction frequencies. Although we did not measure the energetic costs directly, work on ensiferan insects has shown that acoustic signalling involving rapid and intense stridulatory activity is associated with increased metabolic expenditure and elevated thoracic temperature (Erregger et al., 2017). This supports the idea that fast, high-power stridulation is energetically expensive, even if the exact costs in *Agnothecous* remain to be quantified.

In larger species, retaining the ancestral *harmonic amplification* mechanism would reduce energetic cost, but would also lead to lower dominant frequencies due to size-related constraints. Several non-exclusive selective pressures may therefore favour the maintenance of high-frequency calls despite their likely energetic demands. High frequencies may reduce acoustic competition with sympatric species calling in the 4–8 kHz range (*e.g Koghiella* Otte, 1987; *Notosciobia* Chopard, 1915; Anso et al., 2016). In addition, calling songs primarily serve to attract females, and their evolution is constrained by female auditory sensitivity. In Lebinthini, transitions to high-frequency calling are associated with reduced sensitivity to low-frequency components (ter Hofstede et al., 2015). In this context, maintaining high frequencies in *A. tapinopus* and *A. robustus* may reflect female auditory adaptation to high-frequency signals, preventing a return to low-frequency communication, even when body size increases. These scenarios currently remain hypotheses that require direct experimental testing.

These considerations suggest multiple compensatory strategies. Larger species may invest more energy to maintain high-frequency output. Another possibility is the irreversibility of high-frequency evolution: species may rarely, if ever, switch back from high to low frequencies, even at the scale of sister species, despite the behavioural flexibility of the stridulatory system.

At a mechanistic level, the *harmonic amplification* mechanism, likely originating ∼50 Ma in Lebinthini (Tan et al., 2021), is characterised by a mismatch between tooth strike rate and dominant frequency. This implies that harmonic dominance emerges during the amplification stage, when the vibratory signal from stridulation interacts with the resonant properties of the forewing membrane. Acquisition of dominant harmonics in Lebinthini may therefore reflect biophysical adaptations of the forewings that preferentially amplify high-frequency components of the stridulatory signal, a phenomenon also observed in rare individuals of *Oecanthus nigricornis* Walker, 1869 (Sismondo, 1979). In *Agnothecous*, convergent shifts from *harmonic amplification* to *high-speed stridulation*, suggest that behavioural traits such as wing speed are more labile than the resonant properties of the forewings, and can evolve rapidly to match new resonant modes.

### Disparity of calling songs and the interplay of morphology and behaviour on signal evolution

Beyond the two high-speed stridulating species, *A. tapinopus* and *A. robustus*, substantial acoustic diversity exists across *Agnothecous*, even among species sharing the *harmonic amplification* mechanism. Syllable duration range from 10.4 ms in *A. sarramea* to 20.1 ms in *A. albifrons*, and dominant frequency from 13.1 kHz in *A. sarramea* to 18.6 kHz in *A. clarus* (Table 2). As in *A. tapinopus* and *A. robustus*, this diversity reflects the combined effects of morphological variation (primarily body size) and behavioural variation, particularly the introduction of silent intervals within syllables.

Ancestral state reconstructions reveal no consistent evolutionary trend in dominant frequency (Fig. 4). Species with the highest frequencies (*A. clarus*; *A.chopardi*; *A. brachypterous* Gorochov, 1986; *A. pinsula*) are also the smallest (Figs. 4A and 4B), yet no significant correlation between dominant frequency and body size was detected (Fig. 3). Such correlations, however, are being well documented in animals, including crickets (*e.g.*, Jacot et al., 2005; Ower, Hunt & Sakaluk, 2017; Scheuber, Jacot & Brinkhof, 2003; Simmons & Ritchie, 1996; Simmons, 1995; Simmons & Zuk, 1992; Webb & Roff, 1992). The use of amplified harmonics may weaken this relationship, as it partially decouples the dominant call frequency from the carrier frequency generated by stridulation.

By contrast, temporal traits show strong phylogenetic structure. Syllable duration (Fig. 4) clearly distinguishes two major clades (clade A and B with rather long and short syllables, respectively; Fig. 2). Analyses of corrected syllable duration and sound ratio show that these differences primarily result from the insertion of silent intervals caused by discontinuous forewing closure during stridulation (Montealegre-Z, Morris & Mason, 2006). Acoustic analyses across species and a high-speed video analysis of *A. obscurus* (Video S1) confirm the occurrence of such pauses in *Agnothecous*. Similar mechanisms are known in other Lebinthini (*e.g.*, *Lebinthus* Stål, 1877; Robillard et al., 2013) and in tettigoniids (Montealegre-Z, Morris & Mason, 2006), where pauses allow ultrasonic pulse trains by locking the plectrum behind a tooth, storing elastic energy, and releasing it at high speed (Montealegre-Z, Morris & Mason, 2006; Morris, 1970; Morris & Pipher, 1972). Silent intervals lengthen syllables, compensating for the shortening effect of high stridulation speeds and influence female perception (Robillard et al., 2013; Fig. S2), but they also reduce tonal quality. Their adaptive significance in Lebinthini remains unclear and likely involves complex male–female interactions. Notably, *A. pinsula* shows a convergent emergence of this behaviour, with a low sound ratio (0.58) despite belonging to clade B.

The combined effects of morphology and behaviour are particularly clear in the sister species *A. brachypterus* and *A. minoris* (species 11 and 12 in Fig. 4). Both share the *harmonic amplification* mechanism and similar size (body size = 3.20 mm in *A. brachypterus*, 2.80 mm in *A. minoris*; Table 2). Yet their dominant frequencies differ markedly: 18.08 kHz in *A.brachypterus versus* 13.52 kHz in *A. minoris*. The difference is not explained by wing speed alone (87 mm s^−1^ *vs.* 77 mm s^−1^; Table 2). Instead, morphological traits differ: *A. brachypterus* has more functional stridulatory teeth (90 *vs.* 80) and a smaller inter-tooth distance (11.12 μm *vs.* 12.28 μm; Table 2), resulting in a higher tooth strike rate (∼7,800 *vs.* 6,300 teeth s^−1^). These differences account for much of the frequency divergence. Their calls also differ in syllable duration: 15.96 ms in *A. brachypterous versus* 12.46 ms in *A. minoris*. The sound ratio shows that *A. brachypterus* introduces silent intervals (sound ratio = 0.72), while *A. minoris* does not (sound ratio = 1.00; Table 2). This example illustrates how morphological and behavioural traits combine to generate acoustic diversity, even among sister species, shaping both spectral and temporal features of their calls.

## Conclusion

We investigated how behavioural and morphological traits interact to generate acoustic signal diversity in closely related species of the genus *Agnothecous*. Our central hypothesis was that similar high-frequency calling phenotypes can arise through distinct evolutionary routes involving behavioural changes in stridulation dynamics, morphological changes in wing resonance, or both.

Our results support this hypothesis. We show that *Agnothecous* species achieve high-frequency calls through two fundamentally different mechanisms. Most species rely on *harmonic amplification*, in which a multiple integer of the low carrier frequency is amplified to produce a dominant harmonic *via* the resonant properties of the harp. In contrast, *A. robustus* and *A. tapinopus* generate high-frequency calls through *high-speed stridulation*, where the dominant frequency directly matches the tooth-strike rate. Phylogenetic reconstructions indicate that *harmonic amplification* is ancestral in the genus and that *high-speed stridulation* evolved independently at least twice. These shifts are associated with strong increases in wing-closing speed and tooth-strike rate, highlighting the key role played by behavioural innovation.

Our analyses also reveal a tightly integrated sound-production mechanism in which morphological, behavioural and acoustic traits evolve in concert. This demonstrates that signal evolution cannot be reduced to morphology or behaviour alone but emerges from their interaction.

Several limitations remain. Direct kinematic measurements are available for only a subset of species, and unsampled taxa may reveal additional diversity. Future work should combine broader kinematic datasets, biomechanical modelling of wing resonance, and studies of female preferences and ecological context. More speculatively, different sound-production strategies may reflect alternative adaptive solutions to sexual and ecological selection.

##  Supplemental Information

10.7717/peerj.21036/supp-1 Supplemental Information 1 Measured inter-tooth distances (ITD) for each male individual sampled in the 14 Agnothecous species studied (data for *Agnothecous robustus* are absent, and were extracted from Robillard et al., 2013) ITD were measured between successive teeth on the length of the file, from anal to basal. The active part of the stridulatory file considered in the study for each species is indicated in red.

10.7717/peerj.21036/supp-2 Supplemental Information 2 Scanning electron microscope photographs of the stridulatory file for 14 species of *Agnothecous* and tooth distribution on the active stridulatory files (right forewing) Shown are the inter-tooth distances (ITD) between successive teeth on the length of the file from anal to basal. The active part of the stridulatory file considered in the study for each species is framed in red.

10.7717/peerj.21036/supp-3 Supplemental Information 3 Waveform and spectral structure of syllables with and without silent intervals in *Agnothecous.* Waveform representations of calling songs from three *Agnothecous* species illustrating differences in temporal and spectral structure. (A) *Agnothecous tapinopus*, a species producing continuous syllables without silent intervals. (B-C) *Agnothecous azurensis* and *Agnothecous meridionalis*, species exhibiting silent intervals within the syllable. For each species, the structure of the echeme is shown, together with the syllable including silent intervals (beige shading) and the corresponding ”corrected” syllable obtained after removing silent intervals. The frequency spectrum of the corrected syllable is shown for each species, with the main frequency peaks highlighted. TSR = tooth strike rate.

10.7717/peerj.21036/supp-4 Supplemental Information 4 Ancestral states reconstructions for the harp surface (A), file length (B), inter-tooth distance (C) and number of functional teeth (D) analysed in the 15 sampled species in *Agnothecous* Variation of each trait value along phylogeny branches is indicated with a colour scale. Traits values for the 15 tip species are indicated with a coloured circle. Reconstructed ancestral values at the base of each phylogeny is indicated.

10.7717/peerj.21036/supp-5 Supplemental Information 5 Detailed acoustic sampling of the 15 *Agnothecous* species analysed

10.7717/peerj.21036/supp-6 Supplemental Information 6 Molecular data used in the phylogenetic analyses, extracted from (Nattier et al., 2012)

10.7717/peerj.21036/supp-7 Supplemental Information 7 Complete list of 104 specimen accessions at Xeno-canto

## Additional Information and Declarations

### Field Study Permissions

The following information was supplied relating to field study approvals (*i.e.*, approving body and any reference numbers): The research and collection permits have been granted by the Directions de l’Environnement of New Caledonia’s Province Nord and Province Sud for permits to work in natural parks and forest reserves.

### Data Availability

The following information was supplied regarding data availability: Raw data is available in the Supplemental Files and at FigShare: - Gaiddon, Teddy; Nattier, Romain; Legendre, Frédéric; Goutte, Sandra; S. C. Yotoko, Karla; Robillard, Tony (2025). Supplemental Information - Behaviour-structure interplay drives acoustic signal divergence: emergence of multiple mechanisms in closely related crickets. figshare. Online resource. https://doi.org/10.6084/m9.figshare.30329401.v3
The specimens are available at Xeno-canto: https://xeno-canto.org/
- the complete list of 104 accession numbers is available in the Supplemental Files.
