# Supplementary figures and images for: Behaviour-structure interplay drives acoustic signal divergence: emergence of multiple mechanisms in closely related crickets
Source: PeerJ. 2026 Jun 10;14:e21036. doi: 10.7717/peerj.21036 (PMC13264277; doi:10.7717/peerj.21036)

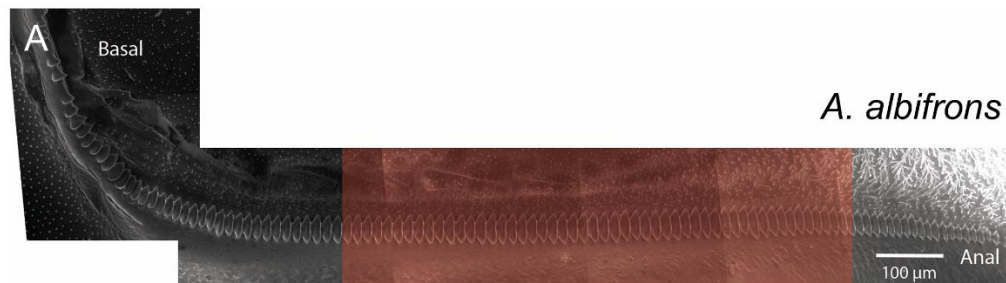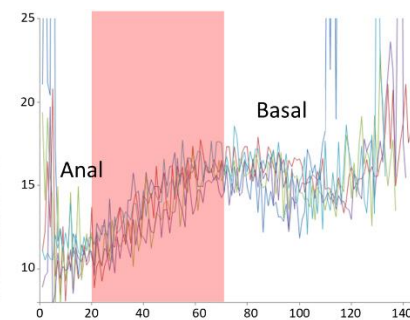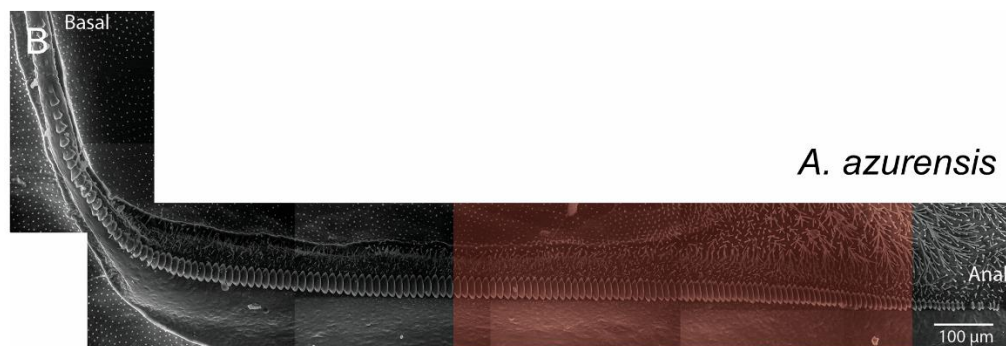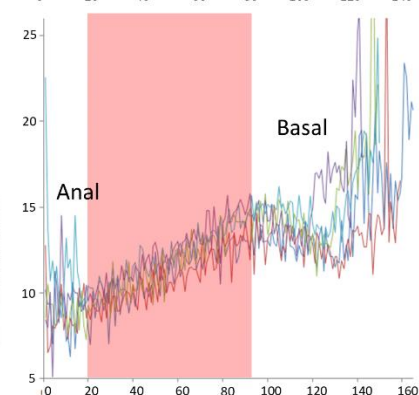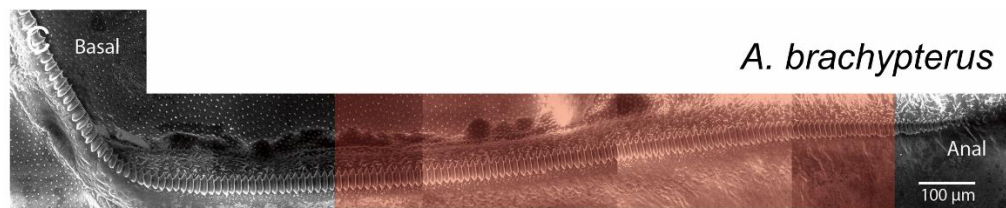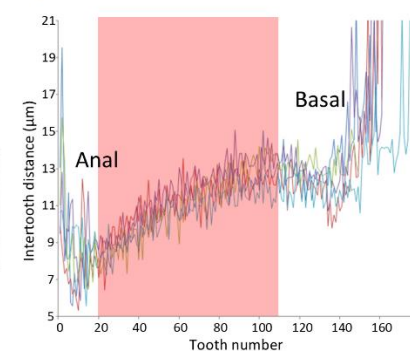

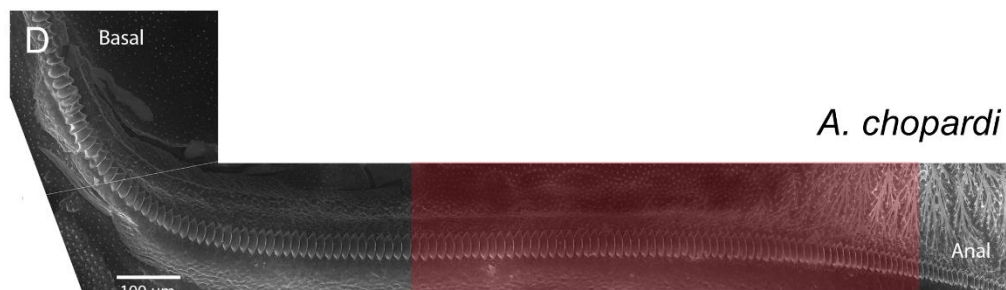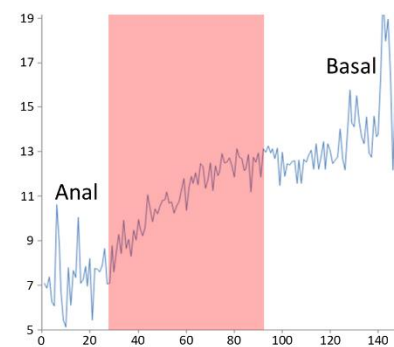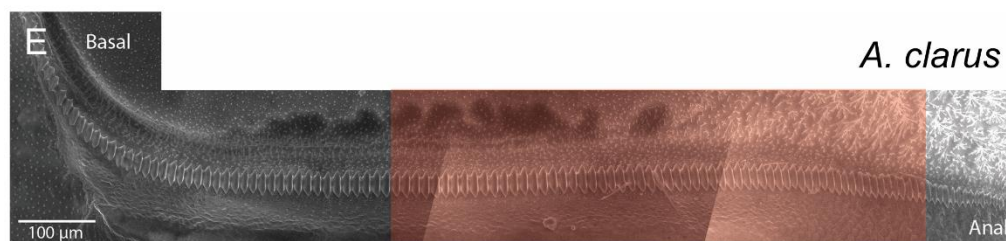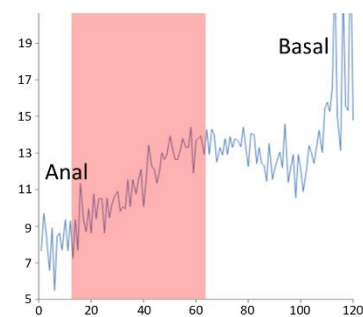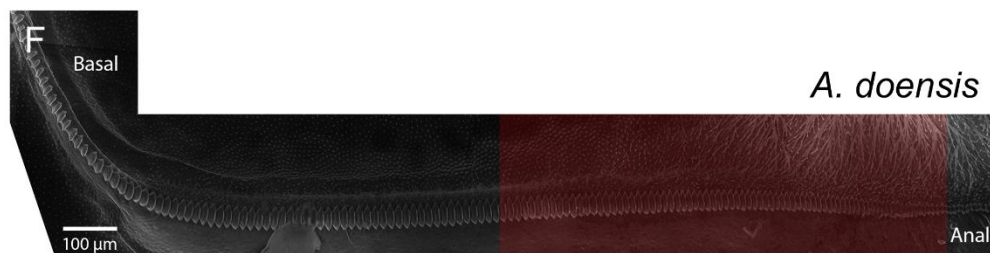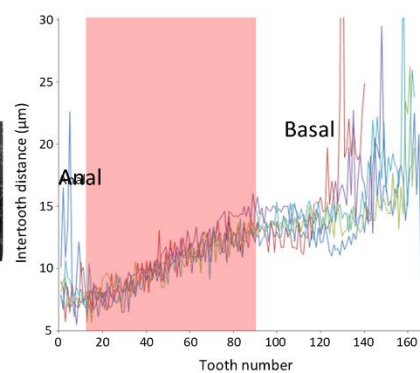

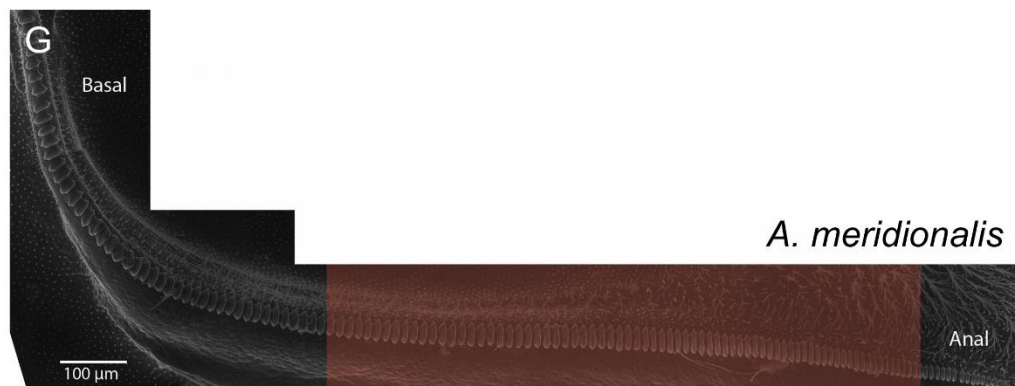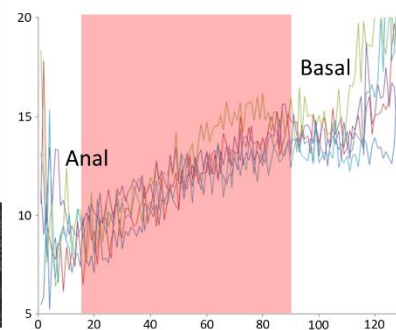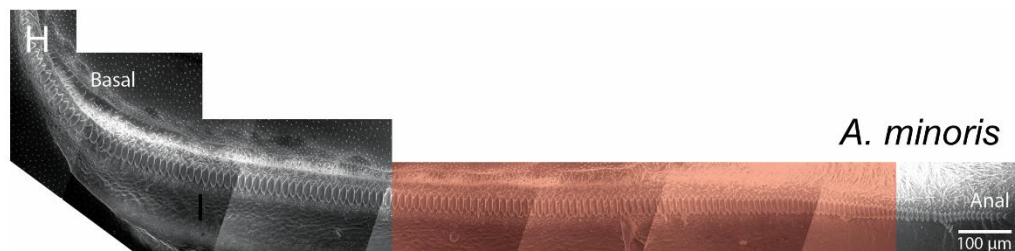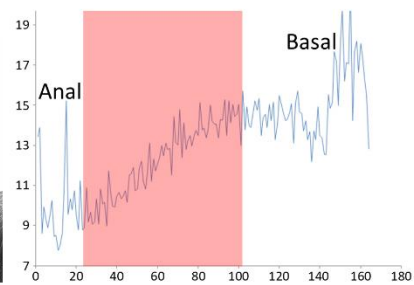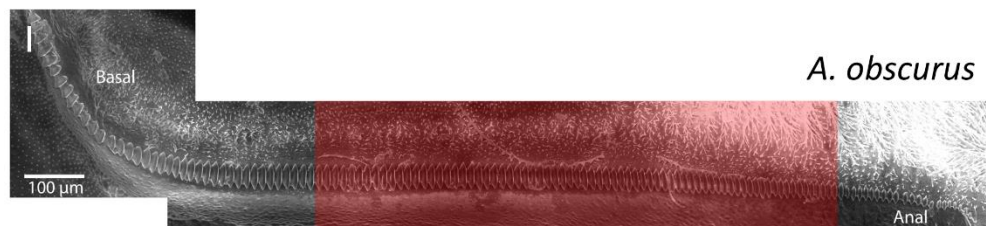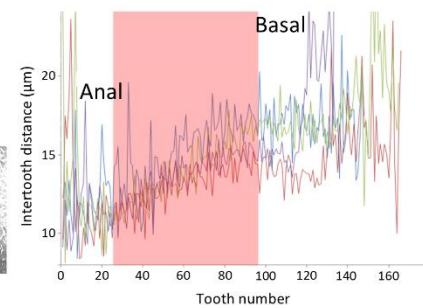

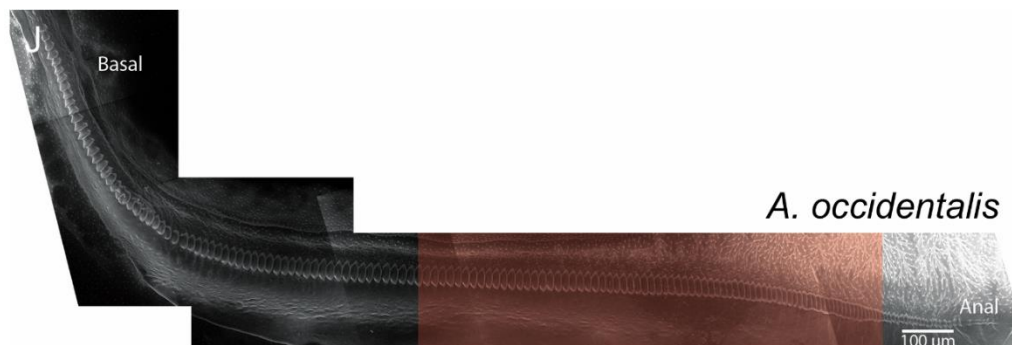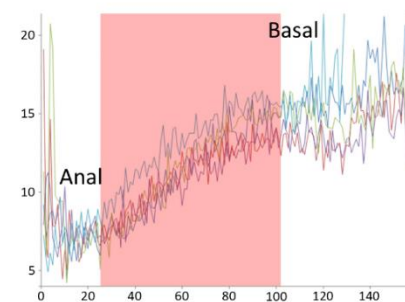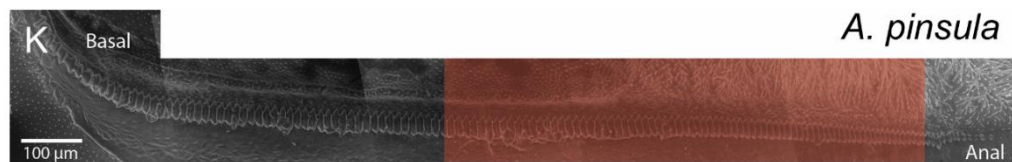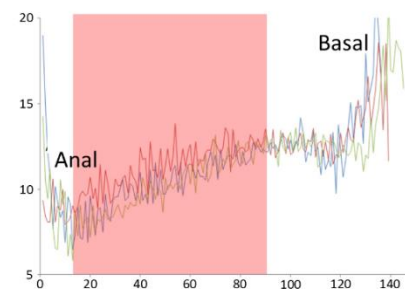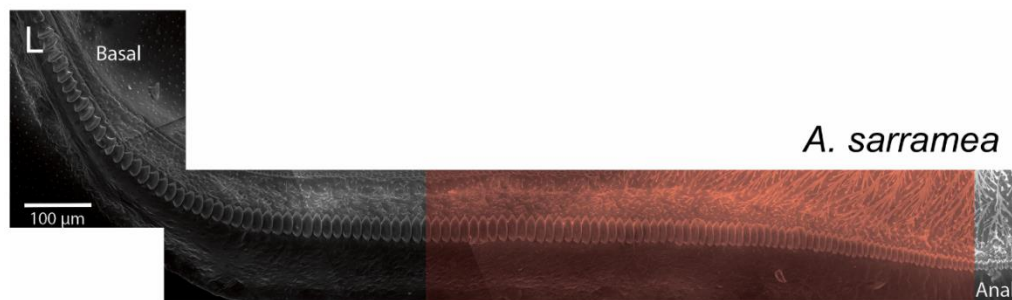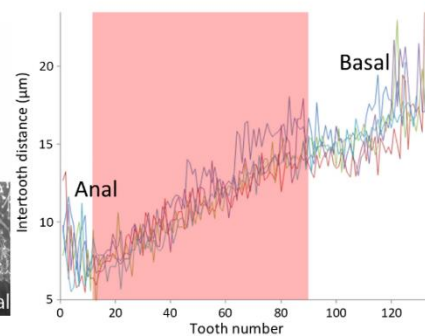

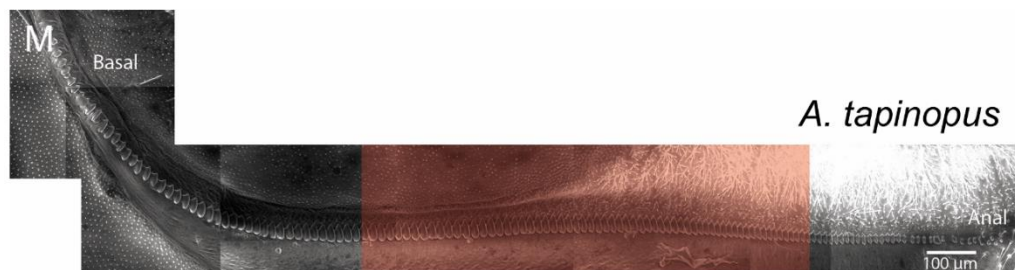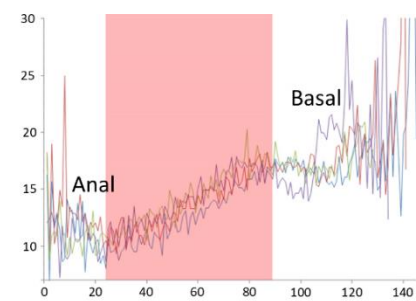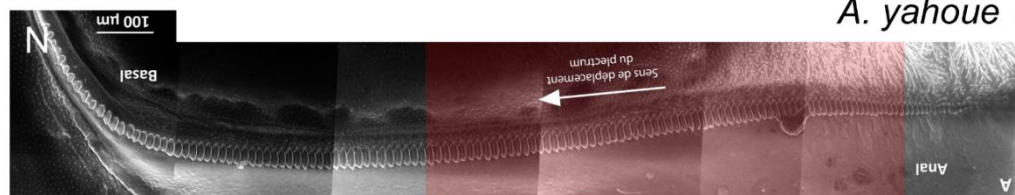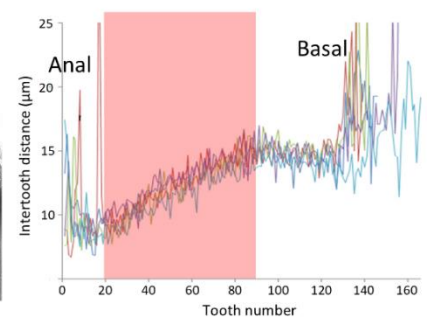

Supplement: Supplemental Information 2 — Shown are the inter-tooth distances (ITD) between successive teeth on the length of the file from anal to basal. The active part of the stridulatory file considered in the study for each species is framed in red. [file peerj-14-21036-s002.pdf]

*Agnothecous tapinopus*

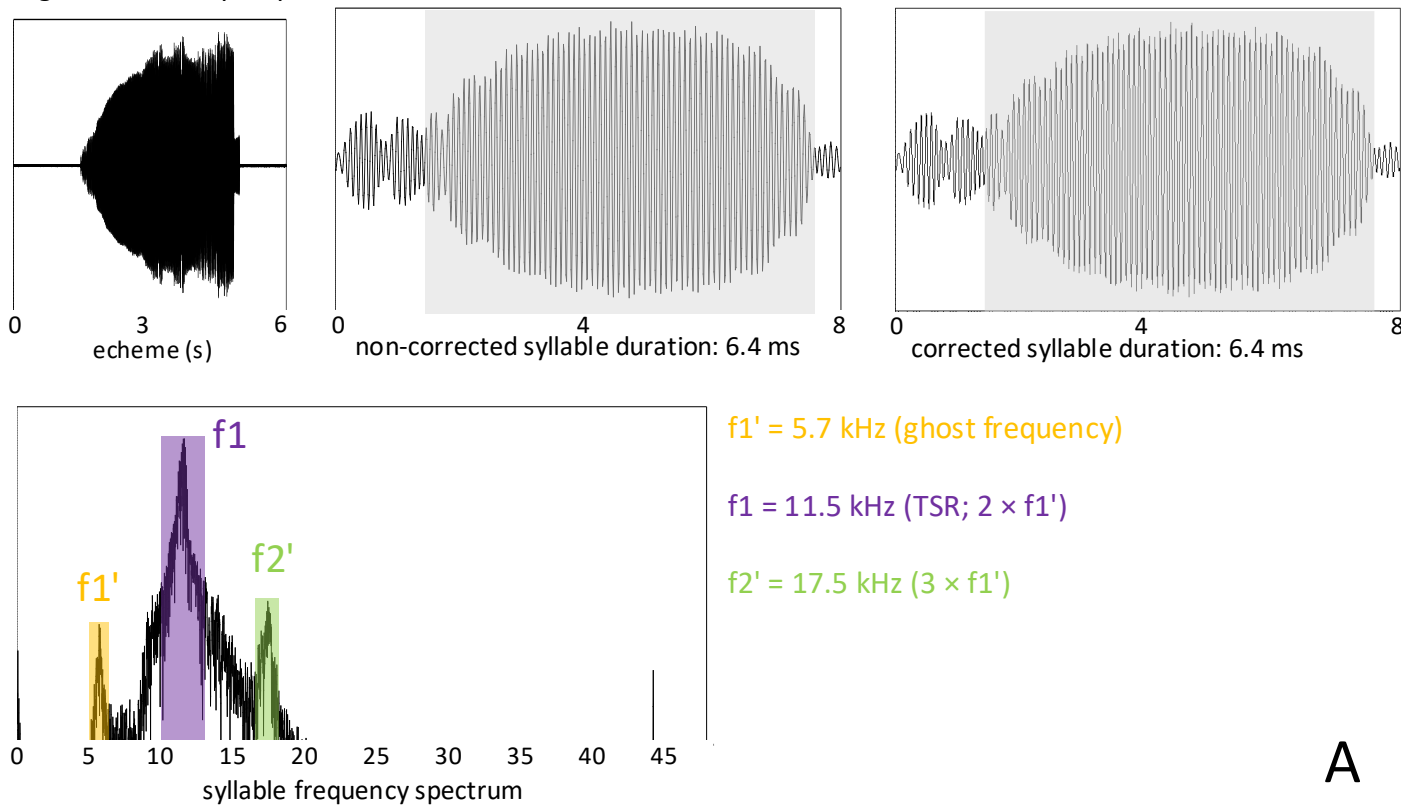

A

*Agnothecous azurensis*

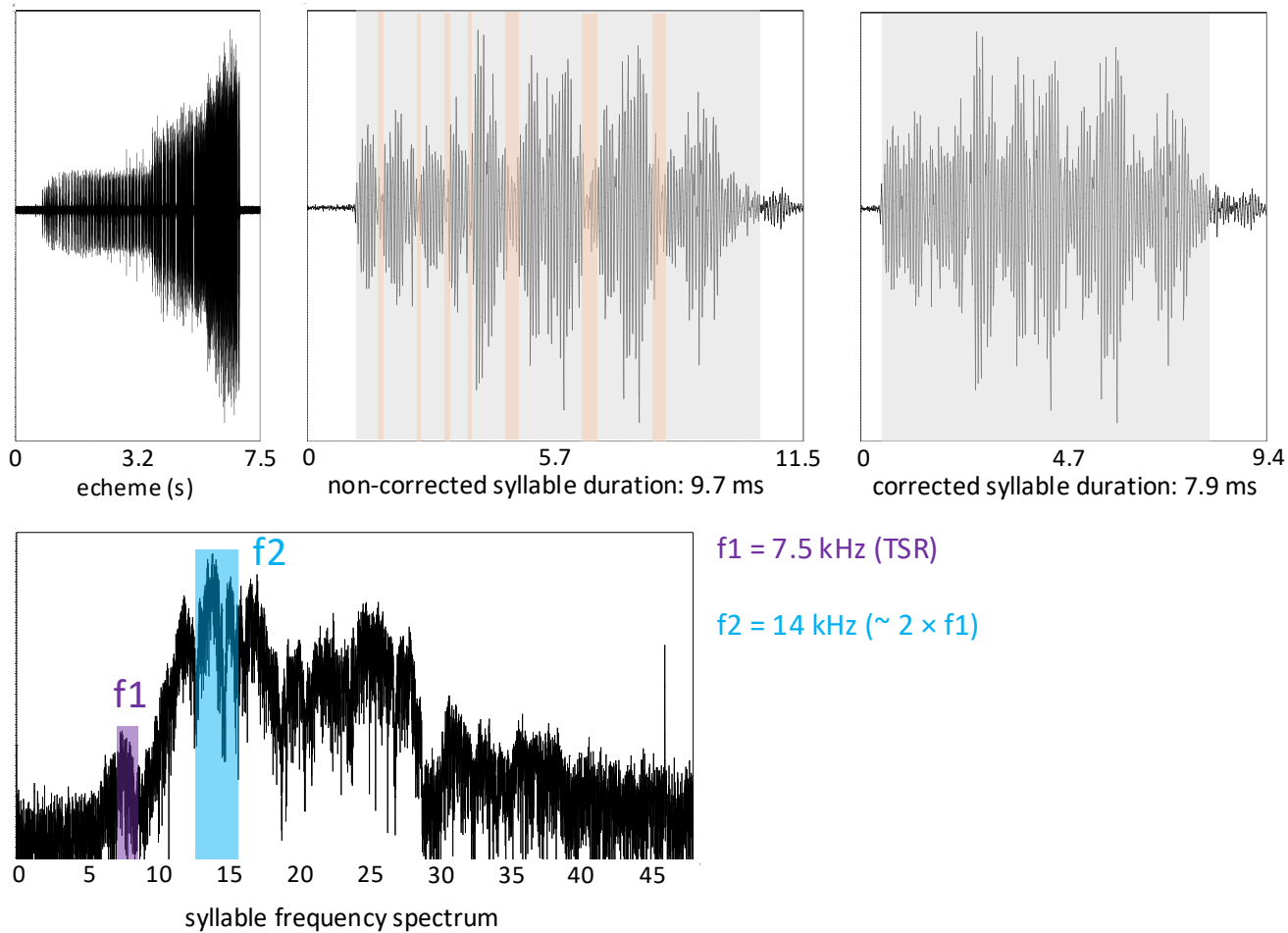

B

*Agnothecous meridionalis*

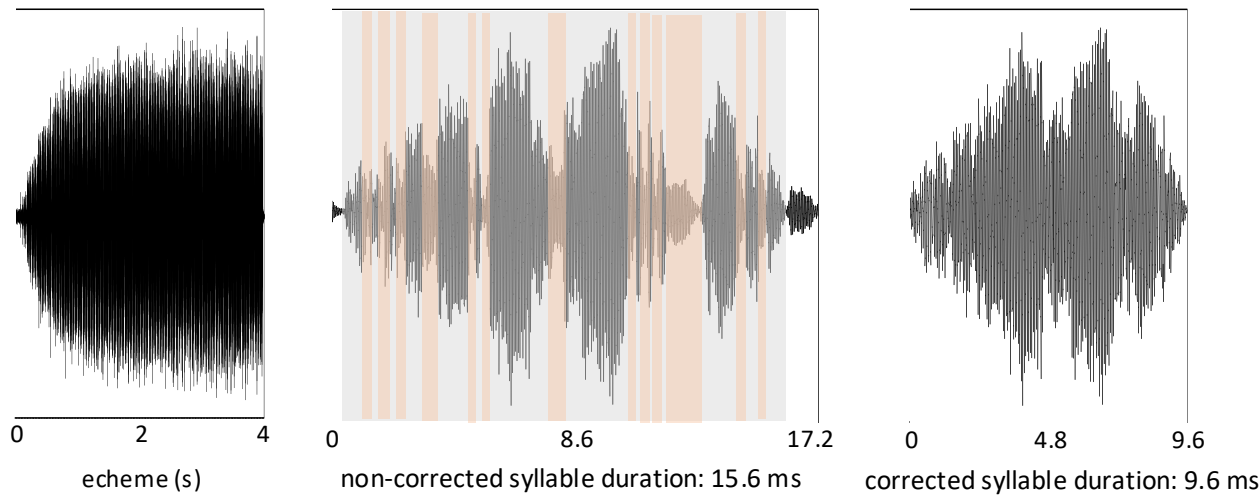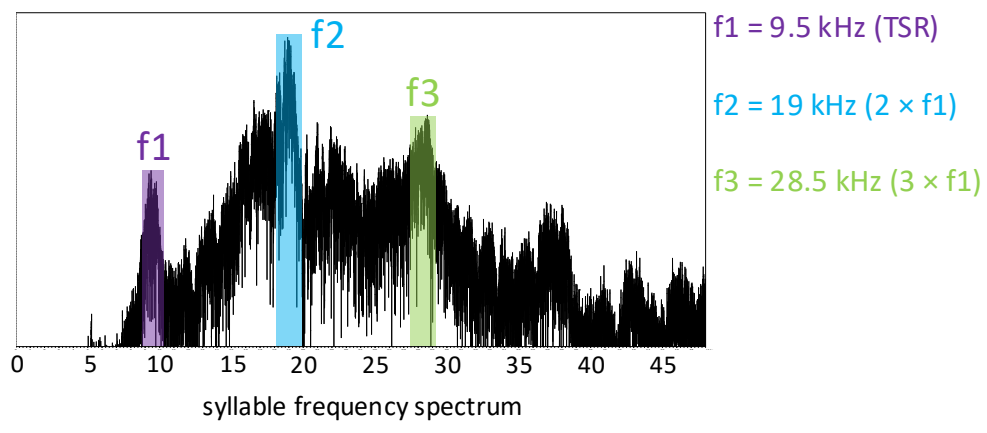

C

Supplement: Supplemental Information 3 — Waveform representations of calling songs from three Agnothecous species illustrating differences in temporal and spectral structure. (A) Agnothecous tapinopus, a species producing continuous syllables without silent intervals. (B-C) Agnothecous azurensis and Agnothecous meridionalis, species exhibiting silent intervals within the syllable. For each species, the structure of the echeme is shown, together with the syllable including silent intervals (beige shading) and the corresponding ”corrected” syllable obtained after removing silent intervals. The frequency spectrum of the corrected syllable is shown for each species, with the main frequency peaks highlighted. TSR = tooth strike rate. [file peerj-14-21036-s003.pdf]

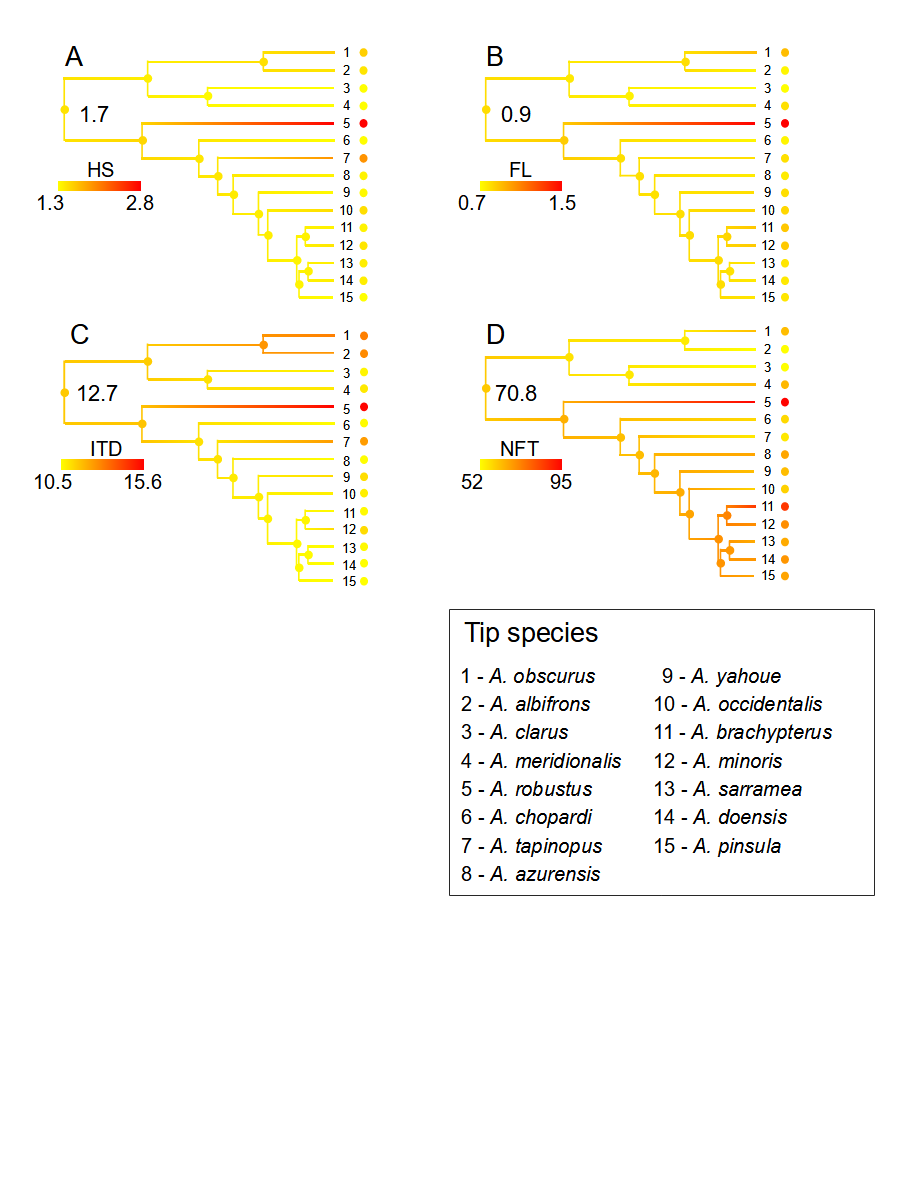

Supplement: Supplemental Information 4 — Variation of each trait value along phylogeny branches is indicated with a colour scale. Traits values for the 15 tip species are indicated with a coloured circle. Reconstructed ancestral values at the base of each phylogeny is indicated. [file peerj-14-21036-s004.png]
