# Supplementary material for: Behaviour-structure interplay drives acoustic signal divergence: emergence of multiple mechanisms in closely related crickets
Source: PeerJ. 2026 Jun 10;14:e21036. doi: 10.7717/peerj.21036 (PMC13264277; doi:10.7717/peerj.21036)
Supplement: Supplemental Information 6 [file peerj-14-21036-s006.docx]

|  | |  | GenBank accession number | | | | | | | | | |
| --- | --- | --- | --- | --- | --- | --- | --- | --- | --- | --- | --- | --- |
|  |  | **Voucher specimens** | **MITOCHONDRIAL** | | | | | **NUCLEAR** | | | | |
| Species | **Locality** | **MNHN collection number** | **16S** | **12S** | **Cytb** | **CO1** | **CO2** | **28S** | **EF1a** | **Hexk** | **H3** | **18S** |
| *Acheta domesticus* (Linnaeus, 1758) | *Worldwide distribution* | *-* | AF248698 | ADZ97611 | AF248682 | JX897403 | JX897439 | JX897465 | JX897494 | - | - | AD18SITS1 |
| *Agnothecous albifrons* Desutter-Grandcolas, 1997 | *Gelima* | *MNHN-ENSIF-1771* | JX897354 | JX897396 | JX897316 | JX897416 | JX897445 | - | JX897529 | - | JX897574 | JX897585 |
|  | *Farino* | *MNHN-ENSIF-1766* | JX897351 | JX897395 | JX897315 | JX897417 | JX897444 | - | JX897526 | - | JX897575 | JX897584 |
|  | *Table Unio* | *MNHN-ENSIF-1770* | JX897352 | JX897397 | JX897317 | JX897419 | JX897447 | - | JX897528 | - | JX897573 | JX897581 |
|  | *Col Toma* | *MNHN-ENSIF-2767* | JX897353 | JX897394 | JX897314 | JX897418 | JX897446 | JX897490 | JX897527 | - | JX897572 | JX897583 |
| *Agnothecous azurensis* Desutter-Grandcolas, 2006 | *Pic du Pin* | *MNHN-ENSIF-2778* | JX897361 | JX897390 | JX897332 | JX897427 | JX897455 | JX897473 | JX897506 | - | JX897568 | JX897594 |
|  | *Rivière Bleue (Pourina)* | *MNHN-ENSIF-2780* | JX897358 | JX897376 | JX897329 | JX897423 | JX897453 | JX897475 | JX897502 | - | JX897566 | JX897595 |
|  | *Rivière Bleue* | *MNHN-ENSIF-2789* | JX897374 | JX897377 | JX897330 | JX897425 | - | JX897476 | JX897504 | JX897539 | JX897567 | JX897600 |
|  | *Rivière Blanche* | *MNHN-ENSIF-2779* | JX897346 | - | JX897328 | JX897424 | - | JX897474 | JX897503 | JX897532 | JX897569 | - |
|  | *Grand Kaori* | *MNHN-ENSIF-2777* | JX897360 | JX897389 | JX897331 | JX897426 | - | JX897472 | JX897505 | - | JX897565 | JX897593 |
| *Agnothecous brachypterus pocquensis*  Desutter-Grandcolas, 2010 | *Pocquereux* | *MNHN-ENSIF-2664* | JX897370 | JX897382 | JX897341 | JX897432 | JX897459 | JX897485 | JX897516 | JX897530 | JX897571 | JX897596 |
| *Agnothecous chopardi* Desutter-Grandcolas, 2006 | *Haute Rivière bleue* | *MNHN-ENSIF-2781* | JX897363 | JX897391 | JX897335 | JX897411 | JX897440 | JX897469 | JX897499 | JX897537 | JX897559 | JX897601 |
| *Agnothecous clarus* Desutter-Grandcolas, 2006 | *Rivière Blanche* | *MNHN-ENSIF-2763* | JX897348 | - | JX897325 | JX897408 | - | JX897486 | JX897521 | - | JX897552 | - |
|  | *Grand Kaori* | *MNHN-ENSIF-2776* | JX897343 | - | JX897323 | JX897409 | - | JX897491 | JX897522 | JX897543 | JX897551 | - |
|  | *Pic du Pin* | *MNHN-ENSIF-2788* | JX897347 | JX897400 | JX897324 | JX897407 | JX897451 | JX897492 | JX897523 | JX897544 | JX897554 | JX897590 |
| *Agnothecous doensis* Desutter-Grandcolas, 2006 | *Mont Do* | *MNHN-ENSIF-2782* | JX897368 | JX897381 | JX897340 | JX897431 | JX897460 | JX897480 | JX897513 | JX897536 | JX897558 | JX897592 |
| *Agnothecous meridionalis* Desutter-Grandcolas, 2006 | *Port Boisé* | *MNHN-ENSIF-2771* | JX897350 | JX897402 | JX897313 | JX897410 | JX897442 | JX897489 | JX897520 | - | JX897550 | JX897597 |
|  | *Ile des Pins* | *MNHN-ENSIF-2772* | JX897349 | JX897401 | JX897311 | JX897420 | - | JX897488 | JX897519 | JX897545 | JX897553 | JX897579 |
| *Agnothecous minoris* Robillard, 2010 | *Mont Mou (basis)* | *MNHN-ENSIF-1389* | JX897373 | - | - | JX897428 | - | JX897483 | JX897515 | JX897534 | JX897564 | - |
| *Agnothecous obscurus* (Chopard, 1970) | *Aoupinié* | *MNHN-ENSIF-2786* | JX897356 | JX897398 | JX897319 | JX897415 | JX897449 | - | JX897510 | - | - | JX897591 |
|  | *Mandjelia* | *MNHN-ENSIF-2785* | JX897357 | JX897393 | JX897320 | JX897412 | JX897450 | JX897487 | JX897525 | JX897531 | JX897576 | JX897587 |
|  | *Touho* | *MNHN-ENSIF-2784* | JX897355 | JX897399 | JX897321 | JX897413 | - | - | JX897524 | - | JX897578 | JX897582 |
|  | *Amoa* | *MNHN-ENSIF-2783* | - | JX897392 | JX897318 | JX897414 | JX897448 | - | JX897497 | - | JX897577 | JX897586 |
| *Agnothecous occidentalis* Desutter-Grandcolas, 2006 | *Col des Roussettes* | *MNHN-ENSIF-2765* | JX897362 | JX897386 | JX897322 | JX897434 | JX897461 | - | JX897512 | JX897533 | JX897570 | JX897589 |
| *Agnothecous pinsula* Robillard, 2010 | *Ile des Pins* | *MNHN-ENSIF-2624* | JX897369 | JX897383 | JX897338 | JX897429 | JX897457 | JX897484 | JX897509 | - | JX897562 | JX897599 |
| *Agnothecous robustus* (Chopard, 1915) | *Aoupinié* | *MNHN-ENSIF-2752* | JX897359 | JX897375 | JX897333 | JX897406 | JX897443 | - | JX897498 | JX897535 | JX897555 | JX897588 |
| *Agnothecous sarramea* Desutter-Grandcolas, 1997 | *Mé Aréto* | *MNHN-ENSIF-2764* | JX897372 | JX897380 | JX897342 | JX897430 | JX897456 | JX897471 | JX897511 | JX897538 | JX897561 | JX897598 |
|  | *Table Unio* | *MNHN-ENSIF-2787* | JX897371 | JX897384 | JX897339 | JX897433 | JX897458 | JX897477 | JX897514 | - | JX897560 | JX897604 |
| *Agnothecous tapinopus* Saussure, 1878 | *Mont Mou* | *MNHN-ENSIF-2769* | JX897345 | JX897379 | JX897326 | JX897421 | JX897452 | JX897468 | JX897508 | JX897541 | JX897557 | JX897580 |
|  | *Monts Koghis* | *MNHN-ENSIF-2770* | JX897344 | JX897378 | JX897327 | JX897422 | JX897454 | JX897470 | JX897507 | JX897540 | JX897556 | JX897605 |
| *Agnothecous yahoue*  Otte, 1987 | *Mont Mou (basis)* | *MNHN-ENSIF-2773* | JX897367 | JX897388 | JX897337 | JX897437 | JX897462 | JX897479 | JX897501 | - | - | - |
|  | *Monts Koghis* | *MNHN-ENSIF-2766* | JX897366 | JX897387 | JX897334 | JX897438 | JX897463 | JX897478 | JX897500 | JX897542 | JX897549 | JX897602 |
| *Eneoptera guyanensis* Chopard, 1931 | *French Guiana* | *MNHN-ENSIF-2741* | AY905301 | AY905272 | AY905355 | JX897404 | - | JX897466 | JX897495 | - | JX897547 | AY905331 |
| *Microbinthus santoensis* (Robillard, 2009) | *Vanuatu, Espiritu Santo* | *MNHN-ENSIF-2437* | JF972527 | JF972511 | JF972495 | JX897405 | JX897441 | JX897467 | JX897496 | - | JX897548 | JF972542 |
| *Nisitrus vittatus*  (Haan, 1844) | *Singapore* | *MNHN-ENSIF-2742* | AY905314 | AY905284 | AY905369 | - | - | - | JX897493 | - | JX897546 | AY905340 |
